# 1-(4-Alkyloxybenzyl)-3-methyl-*1H*-imidazol-3-ium organic backbone: A versatile smectogenic moiety

**DOI:** 10.3762/bjoc.5.62

**Published:** 2009-11-06

**Authors:** William Dobbs, Laurent Douce, Benoît Heinrich

**Affiliations:** 1 Institut de Physique et Chimie des Matériaux de Strasbourg, UMR 7504, CNRS-Université de Strasbourg, BP 43, 23 rue du Loess, F-67034 Strasbourg Cedex 2, France

**Keywords:** crystal liquid, imidazolium salts, ionic liquid, supramolecular arrangement

## Abstract

The merger of ionic liquid and liquid crystal fields, obtained by using the imidazolium ring as a common element, has allowed us to tailor a new set of materials which associate specific functionalities. These functionalities are consequences of the original properties of the component, ionic liquids, liquid crystals and their association in a single compound. The study of this interesting association led us to elaborate environment-flexible cationic architectures from which mesomorphic properties emerge. Moreover, we have also explored the influence of different anions on the mesomorphic properties.

## Introduction

Uniting the properties of ionic derivatives with those of liquid crystals, with their many forms of labile macroscopic ordering, raises interesting prospects [1]. By themselves ionic liquids are organic salts (i.e. totally composed of cations and anions) that are – unlike traditional salts – liquid at or near ambient temperatures. The combination of properties is truly unique: ionic liquids are non-volatile and non-flammable, have high chemical and radiochemical stabilities, tunable electric conductivities, exceptional dissolution properties [2–4]. By varying the cations and anions the physico-chemical properties of ionic liquids can be tuned and specifically optimised for a wide range of applications [5–11]. Although many organic cations (e.g. phosphonium, ammonium, pyridinium, imidazolium) can be used for the design of ionic liquids, none of these cations are as popular as the imidazolium ion.

In particular, the modification of the N,N′ substituents in imidazolium systems is a facile mean of creating various amphotropic liquid crystals. Such imidazolium liquid crystals have especially great potential as ordered reaction media that can impart selectivity in reactions by organising reactants, as scaffolds for the synthesis of nanostructured particles [12–14], in dye-sensitized solar cells [15–16] or also in bio-related science [17–18].

In conclusion, this combination represents a fascinating class of new multifunctional materials [19] in which the organic modifications afforded to the imidazolium central part and the ions’ environment (e.g. ion assemblies) closely govern their melting points and their use [20].

The present article concerns a synthetic route to imidazolium salts, which have been modified to present thermotropic liquid crystal behaviour. In association with different anions and in relation to the chain length incorporated in the methylimidazolium cations, we were able to tune the different transition temperatures and thus obtain stable smectic A phase from ambient temperature to 250 °C (decomposition temperature). The mesomorphic properties were studied by polarizing optical microscopy, differential scanning calorimetry and X-ray diffraction. With the support of dilatometry, models for the lamellar supramolecular arrangement of the salts are proposed and its evolution is discussed as a function of the counter ions’ morphology.

## Results and Discussion

### Synthesis and characterization

**Scheme 1:**
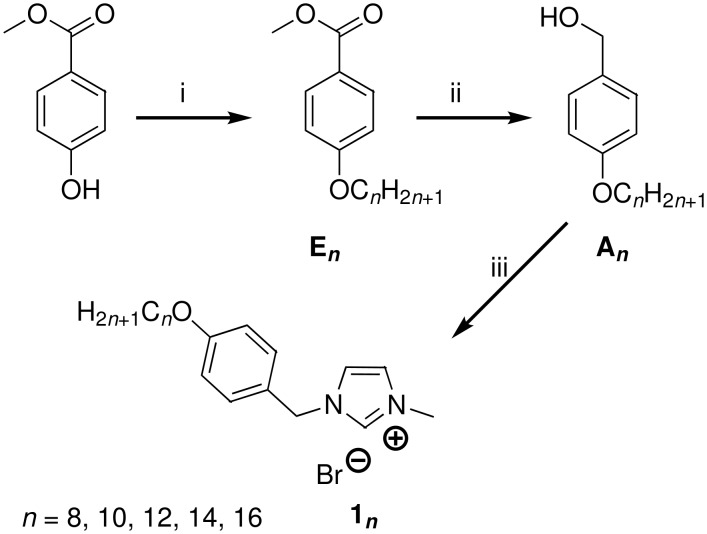
Mesogenic imidazolium synthesis [Reaction conditions: (i) DMF, K_2_CO_3_, BrC*_n_*H_2_*_n_*_+1_, 60 °C, overnight; (ii) LiAlH_4_, THF, Ar, 4 h; (iii) SOBr_2_, DCM, under Ar; THF dry, methylimidazole, inert atmosphere].

Our molecules are based on an organic calamitic structure in which a polar rigid group (methylimidazolium head) is associated to a flexible aliphatic chain with 8 to 16 carbons. The syntheses of all the compounds presented in this study began with the preparation of the bromide imidazolium salts followed by an anionic exchange step. Bromide derivatives **1*_n_*** of 1-(4-alkyloxybenzyl)-3-methyl-*1H*-imidazol-3-ium with *n* = 8, 10, 12, 14, 16 have been synthesized in good yield (>82%) and on the gram scale. These compounds were obtained in three steps, following slightly modified literature procedures. Methyl 4-hydroxybenzoate was etherified with various 1-bromoalkanes (C*_n_*H_2_*_n_*_+1_Br, *n* = 8, 10, 12, 14, 16) in the presence of K_2_CO_3_ in DMF (yield > 96%), and the resulting amphipathic esters **E*_n_*** reduced by LiAlH_4_ to the corresponding benzyl alcohols **A*_n_***. The different resulting alcohols were converted quantitatively into the intermediate 4-(alkoxy)benzyl bromides with thionyl bromide. The final compounds **1*_n_*** were then obtained by quaternization of 1-methylimidazole with the bromides in refluxing THF under inert atmosphere (Scheme 1).

**Scheme 2:**
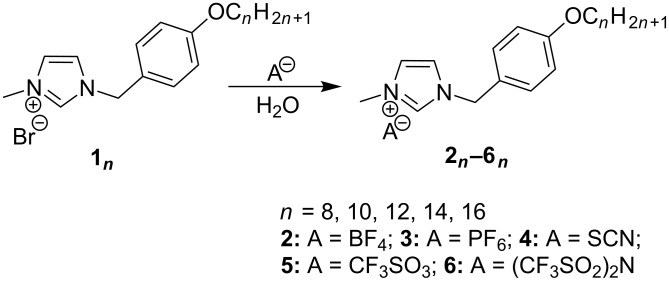
Anion exchange in water.

To obtain the desired set of compounds, we performed as a final step an anionic metathesis in water from **1*_n_*** in order to give **2*_n_***, **3*_n_***, **4*_n_***, **5*_n_*** and **6*_n_*** respectively with BF_4_^−^, PF_6_^−^, SCN^−^, CF_3_SO_3_^−^ and (CF_3_SO_2_)_2_N^−^ anions (Scheme 2) in good yield, easily scaled up to gram quantities. In this part, we used the good solubility of the bromide derivatives **1*_n_*** in water to perform the anionic exchange. By a simple addition in water solution of the corresponding lithium, potassium or sodium salts of the desired products, our targeted compounds were dearly obtained by phase separation (*n* ≤ 10) or precipitation (*n* > 10) according to the chain length.

All imidazolium compounds (**1_8_**–**6_8_**, **1_10_**–**6_10_**, **1_12_**–**6_12_**, **1_14_**–**6_14_**, **1_16_**–**6_16_**) were purified by flash chromatography or crystallization. Their structural characterizations and their purities were established by ^1^H NMR, ^13^C NMR{^1^H}, FT-IR spectroscopy and elemental analysis.

Due to their close chemical structures, similar behaviour was observed during NMR characterization for all **1*_n_*** samples as a function of concentration. A significant chemical shift related to the proton of the imidazolium head (H_im_), and in particular a Δδ = 0.43 ppm (from low to high concentration) for the H_im_ carried by the carbon atom between the two nitrogen atoms, was observed for **1_12_**. As already mentioned in a previous article [21], these spectroscopic results clearly indicate the formation of aggregates in solution and underline the high sensitivity of these protons to their environment.

**Figure 1:**
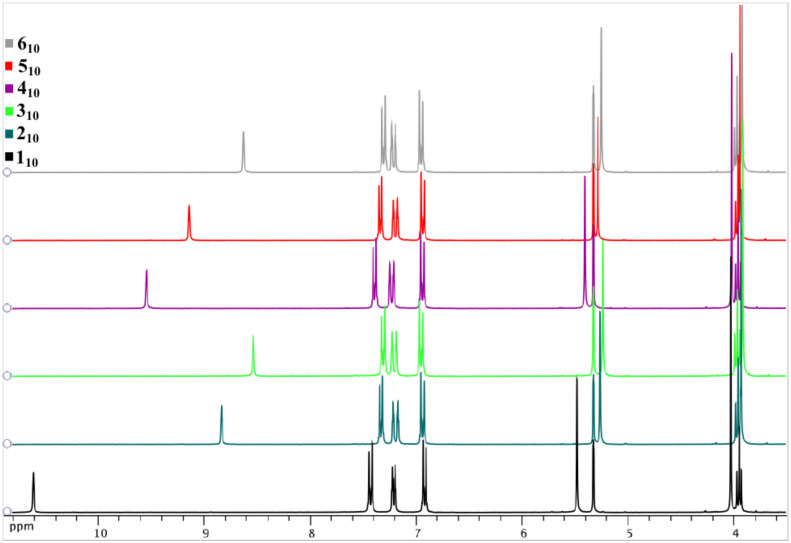
^1^H NMR spectrum (in CD_2_Cl_2_) of **1_10_**–**6_10_**.

This second point was definitively demonstrated by a NMR study performed as a function of the anionic species (constant concentration [*c*] = 3.1·10^−2^ mol·L^−1^) (Figure 1).

Related to their morphologies and to their abilities to interact with the imidazolium moiety, we observed significant variation of the chemical shift of the H_im_ between the two nitrogen atoms: δ = 10.61 ppm (Br^−^); 8.83 ppm (BF_4_^−^); 8.53 ppm (PF_6_^−^); 9.54 ppm (SCN^−^); 9.14 ppm (CF_3_SO_3_^−^); 8.63 ppm ([CF_3_SO_2_]_2_N^−^). We could also observe some modifications of the spectrum for the protons of the first substituent group of the imidazolium core (N–CH_3_ and N–CH_2_) localized respectively around 4 ppm and 5.3 ppm.

Correlated to the crystal structure of compound **1_12_** these observations were made on all the protons used for the organization of the polar part by multiple anion-cation and cation-cation interactions [21]. These NMR studies carried out as a function of the nature of the anion are a reflection of the modifications that occur in the polar layer in function of the anion source in solution.

### Investigation of the liquid crystalline behaviour

As expected most of the ionic compounds showed liquid crystalline properties [19,22–30]. Their characterization was the result of the combination and interpretation of thermal studies (Thermogravimetry Analysis or TGA, Differential Scanning Calorimetry or DSC), direct polarized optical microscopy (POM) observation and small angle X-ray diffraction studies (SAXS).

### Thermic behaviour

**Figure 2:**
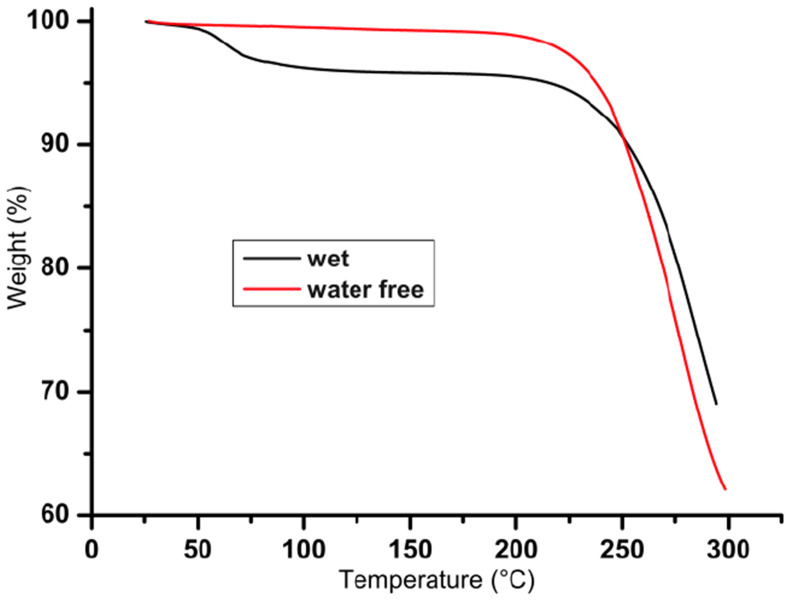
TGA measurements of wet and water free **1_12_** imidazolium salt.

Due to their amphipathic structures, all imidazolium salts interact more or less rapidly with the water contained in air. This property explains the presence of different amounts of water in the elemental analyses. Otherwise, water-free samples can be obtained and used for the investigation of liquid crystalline behaviour, if we conserved them under vacuum at 50 °C during the night preceding the analysis (Figure 2).

**Figure 3:**
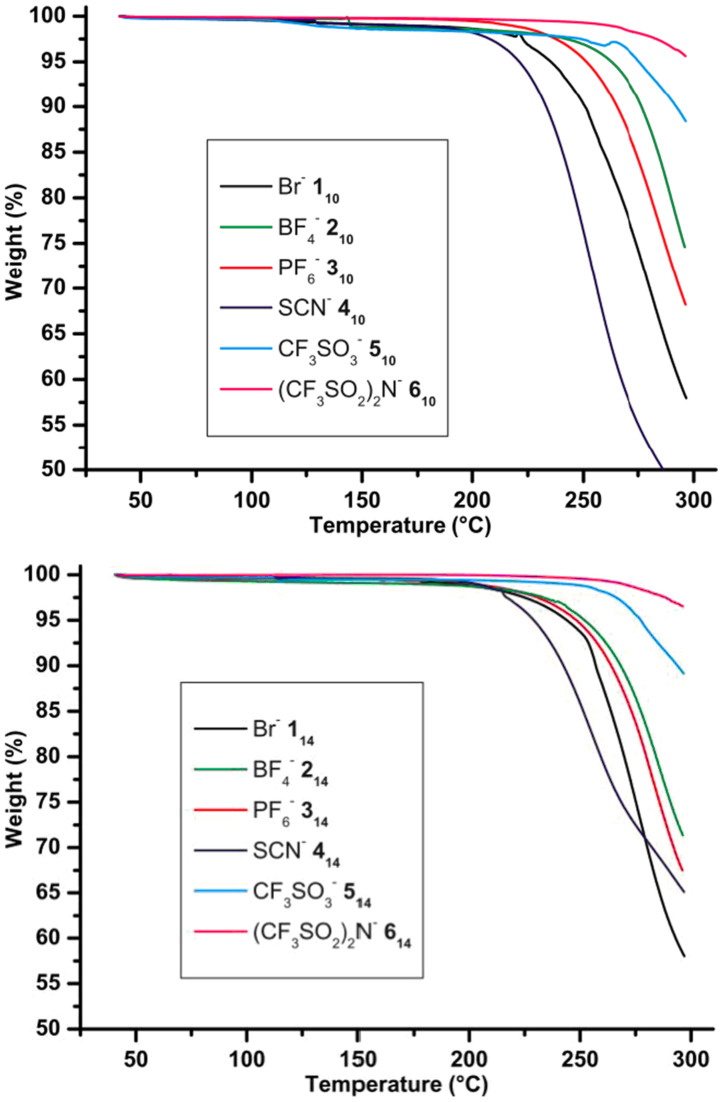
TGA measurements of the two entire series **1_10_**–**6_10_** and **1_14_**–**6_14_** (rate 10° C·min^−1^, in air).

The TGA realized on all samples indicated their good stability; no degradation occurred below 220 °C (Figure 3). The analysis showed a degree of stability as a function of the anion but no real tendency can be extracted as a function of chain length. In all series the general order of stability order is SCN^−^ ≈ Br^−^ < BF_4_^−^ ≈ PF_6_^−^ < CF_3_SO_3_^−^ < (CF_3_SO_2_)_2_N^−^.

To avoid any phenomenon related to the sample preparation (purification, crystallisation, hydration) our reported DSC results are obtained from the second thermic cycle (2^nd^ heating and cooling) and all phase transitions described occurred in the ionic liquids between −80 °C to 200 °C (ESI). The DSC graphs for the compounds with short aliphatic chains exhibit a glass transition during the cooling process rather than a crystallization peak. In contrast, increasing the chain length (*n* > 10) we have almost detected a crystalline transition. During the following heating process, most of these compounds (*n* > 10) show crystalline polymorphism with some rearrangement between different solid organizations (cold crystallization, transition crystal to crystal) (Table 1).

**Table 1:** Temperatures and enthalpy changes of the phase transitions for **1_8–10_** to **6_8–10_**. G: Glass; Cr: solid; SmA: Smectic A phase; I: isotropic liquid; Dec: decomposition.

**1_n_–5_n_**	Phase sequences °C (kJ·mol^−1^)	**2_n_–6_n_**	Phase sequences °C (kJ·mol^−1^)

**1_8_**	G −15.6(–) SmA 155.1(0.4) I I 155(0.45) SmA −30.7(–) G	**2_8_**	G −29.1(–) SmA 71(0.3) I I 69.9(0.4) SmA −36.1(–) G
**3_8_**	G −26.1(–) Cr 34.8(0.15) I I 33.6(0.2) Cr −31.9(–) G	**4_8_**	Cr 33.7(24.3) SmA 50.9(0.35) I I 50.2(0.28) SmA 7.1(25.6) Cr
**5_8_**	G −44.3(–) I I −51.9(–) G	**6_8_**	G −59.4(–) Cr_1_ −14.5(29.9)^a^ Cr_2_ 26.7(36.3) I I (–) G

**1_10_**	G −14.5 (–) SmA 227(–)^b^ I I 227(–)^b^ SmA −23.2(–) G	**2_10_**	G −29.1 (–) Cr_1_ −0.1(17.9)^a^ Cr_2_ 30(18.0) SmA 143.4(0.47) I I 142.5(0.88) SmA −29.7(–) G
**3_10_**	G −26.2 (–) Cr_1_ 7.1(24.2)^a^ Cr_2_ 66.3 (31.5) SmA 93.3(0.4) I I 92.1(1.1) SmA −33.2(–) G	**4_10_**	Cr 32.3(23.8) SmA 116.6(0.7) I I 115.6(1.0) SmA 8(28.0) Cr
**5_10_**	Cr_1_ −13.1 (5.6)^a^ Cr_2_ 5.31(1.4)^a^ Cr_3_ 13.5 (5.9)^a^ Cr_4_ 45.66 (4.9) Cr_5_ 56.37 (30.8) I I −16.7(–) Cr_1_	**6_10_**	Cr_1_ −24.1 (8.7)^a^ Cr_2_ 36.8(51.3) I I −30.7(17.4) Cr_1_

^a^Cold crystallisation, ^b^Transition observed by POM.

**Figure 4:**
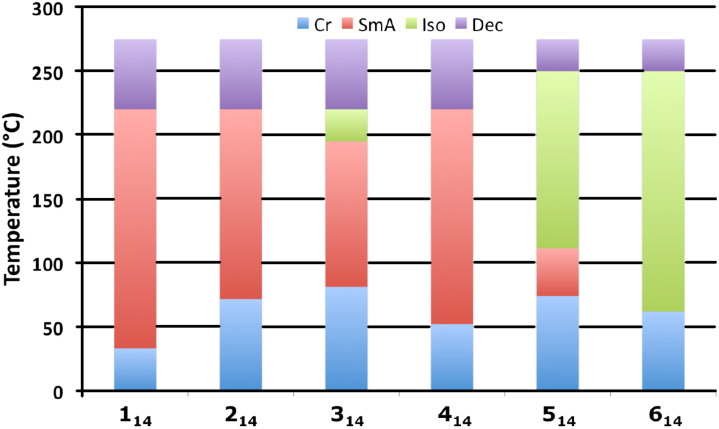
Transition temperatures of **1_14_**–**6_14_** as a function of the anion (Cr = crystal; SmA: smectic A phase; Iso: Isotropic Liquid; Dec: decomposition).

The transitions between crystal and mesomorphic state and also the clearing point increase with the chain length, allowing liquid crystalline properties to exist from sub-ambient temperature (**1_8_**, **2_8_**, **4_8_**) to very high temperature avoiding any isotropization before decomposition of the material (molecules with long alkyl chain *n* ≥ 12). In parallel, the nature of the anion (shape, size) played an important role in the evolution of the clearing point. In the same family (from **1*_n_*** to **6*_n_***) the stability of the liquid crystalline phase dramatically decreases, finally disappearing completely (Figure 4).

This evolution will be more discussed later in regard to the results obtained with the dilatometry study. In summary, only 9 of the compounds did not show liquid crystal characteristics; **6*_n_***, which are constituted by the larger anion (bis[(trifluoromethyl)sulfonyl]amide), are ionic liquid compounds without any supramolecular organization into mesophase. This is also the case in the series of compounds **5*_n_*** with *n* < 12 (only a monotropic mesophase was observed for **5_12_**), and finally for **3_8_**.

### Supramolecular arrangement

**Figure 5:**
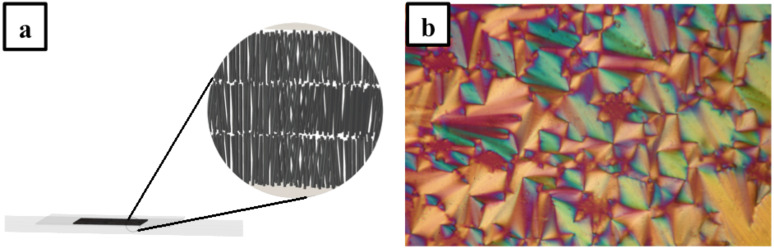
(a) Illustration of a single homeotropic monodomain, which is observed as a black isotropic texture under POM, (b) focal conic texture observed at 80 °C with **1_8_** (spacer Mylar foil 100 μm).

Despite the significant tendency of these molecules to form spontaneously a single homeotropic monodomain (Figure 5a) the nature of the mesophases could be quite easily assigned on the basis of their optical textures if separated microscope slides (100 μm spacer) were used.

The utilization of a spacer allows the formation of large oriented monodomains to be avoided and thus furtive formation of birefringent batonnets upon slow cooling from the isotropic melt, followed by developed focal conic texture (Figure 5b) can be clearly observed. These observations exemplified the existence of a smectic-A phase for all ionic mesomorphic compounds.

**Figure 6:**
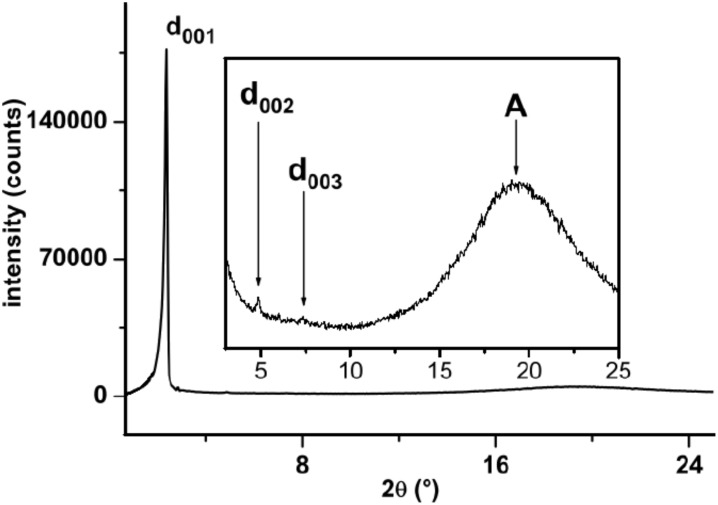
Diffraction small-angle X-ray pattern of the smectic phase of **2_12_** recorded at *T* = 100 °C.

The identification of all mesophases was supported by small-angle X-ray diffraction. SAXS patterns were collected for the mesomorphic salts as a function of temperature (Figure 6). Typically, in all cases, in the large angle region, i.e. at ca. 4.5–4.6 Å, a very intense and diffuse scattering halo was observed, corresponding to the molten alkyl chain in liquid-like order (A), whilst in the low-angle part of the diffractogram one to three equidistant sharp and intense peaks were observed, characteristic of the layered organization.

The very intense first sharp peak was directly correlated to the periodicity value. In each system, the smectic periodicity increases with the chain length and decreases with increasing temperature, reflecting the higher thermal mobility of the alkyl chains, concomitant with the compression of the smectic layers. Interestingly also, the periodicity of the salts is closely correlated to the anion species and the general order is Br^−^ > BF_4_^−^ ≈ SCN^−^ > PF_6_^−^ > CF_3_SO_3_^−^.

### Dilatometric studies

Prior to the analysis of the molecular packing of the various compounds in the smectic mesophase, the molecular volume (*V*_mol_) of one salt, **1_12_**, was measured by using a home built high precision apparatus, incorporating temperature control within 0.03 °C, relative density measurement within 0.01% and absolute density measurement within 0.1%. The molecular volumes of the other homologues and of the analogues with the other counter ions were calculated with an accuracy of 0.5% from the molecular volume measured before and from the methylene and counter ion partial volumes.

Despite the untilted smectic layering and the tiny thermal expansion of the molecular volume mainly due to the contribution of the alkyl tails, all compounds exhibited a very strong dependence upon temperature of the layer spacing. This evidence is the consequence of the evolution of the projection area of a mesogen counter-ion assembly within the mean smectic plane, the thickness of the aliphatic sublayer variating in proportion since the spreading of the liquid alkyl chains occurs without volume variation [31]. This projection area, called "molecular area" S, is equal to the ratio *N*·*V*_mol_/d, in which d is the layer spacing, *V*_mol_ the molecular volume and *N* the number of superposed mesogen counter-ion assemblies in the ionic sublayer. Thus, the alkyl tails expelled from each side of the sublayer containing the ions and the mesogens impose a bilayer (*N* = 2) type of organisation on these sublayers, consisting of two head-to-head facing ionic monolayers.

**Figure 7:**
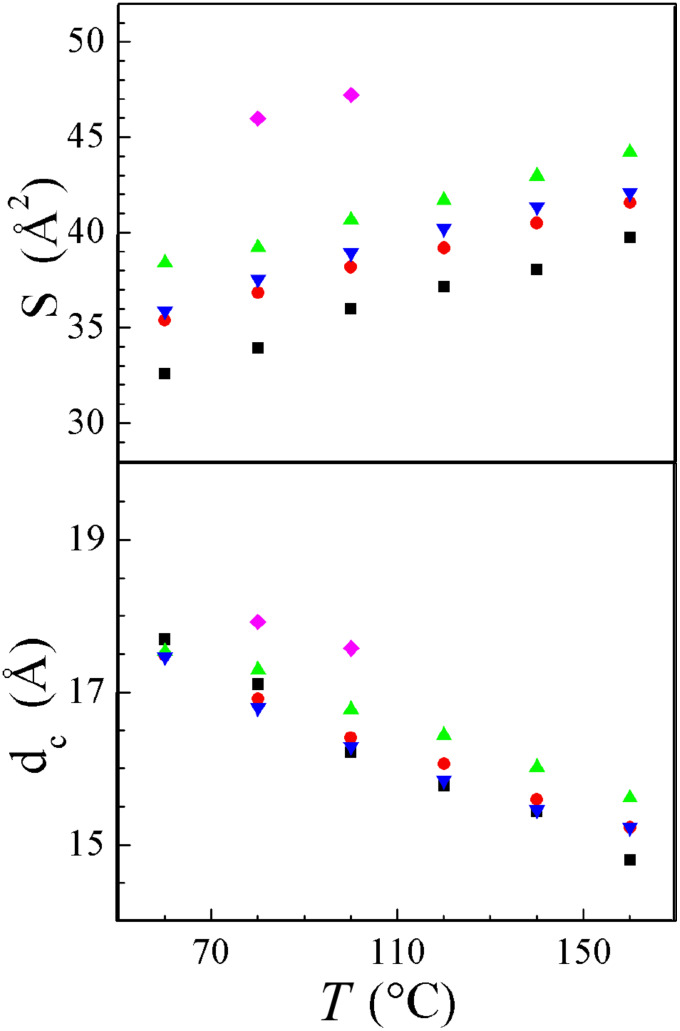
Variation with the counter-ion of the molecular area S and of the ionic sublayer thickness d_c_ (including mesogenic segments) in the smectic A phase for series: squares: **1_14_**; circles: **2_14_**; up triangles: **3_14_**; down triangles: **4_14_**; diamonds: **5_14_**.

The resulting variations of S versus temperature *T* within the analogous series of various anions but constant chain length (see Figure 7) consist of steep increases, approximately linear with very similar slopes and with lateral shifts following the different bulk of the anions. Anion substitution actually leads to large variations of S, but very small variations of the ionic sublayer thickness d_c_ (obtained by subtracting the aliphatic sublayer contribution, see Figure 7), the residual discrepancies being explicable by the different shapes of the anions. This indicates that polymorphic evolution in the series is almost exclusively due an anion size effect with no significant influence on the segregation in sublayers. As a clear confirmation, close to the isotropization temperatures, the values reached by S depend on the chain length but are almost independent of the anion (between 43 and 44 Å^2^ and around 47 Å^2^, for the C_12_ and the C_14_ analogous series respectively, see Table SI-7 in Supporting Information File 1).

**Figure 8:**
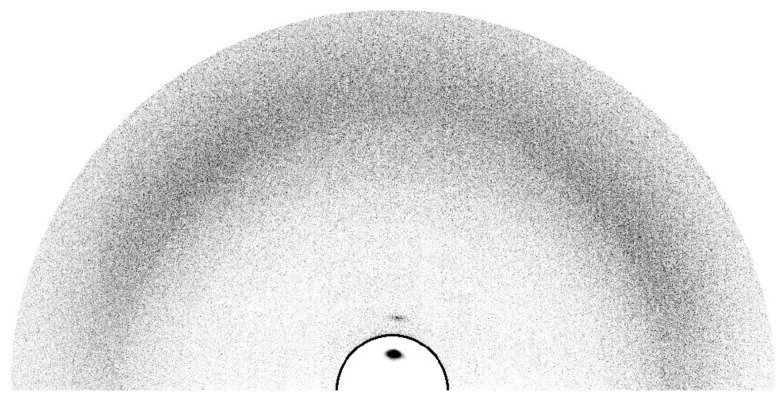
Grazing incidence X-ray pattern at 100 °C on the top of a **3_12_** droplet, slowly cooled down from isotropic phase; for image contrast compatibility with the surrounding zone, intensities inside the zone, delimited by the black line and containing the first order reflection of the lamellar stacking, are divided by one hundred.

So far the results are nearly identical to the ones already discussed for a series with the same counter ions, C_12_ alkyl tails but a small change in the mesogenic part [32]: from S and d_c_ in the smectic A phase and also from comparison with the crystalline organization it was deduced that the enormous difference between the cross-section of the tails and the ionic lattice area is compensated by the gathering of the ionic sublayer and by the folding of the tails. As in the present case, the dependences upon *T* of S and d_c_ indicated the decrease of gathering degree and therefore the increase of folding with increasing *T*. Thus the compromise molecular areas are still 1.5 to 1.9 times larger than the cross-sectional area of a molten but stretched aliphatic chain (between 21 and 24 Å^2^, depending on *T*), from which a high degree of folding geometrically equivalent to 50° random tilting is deduced. Indeed, this significant folding of the tails is directly observed in oriented X-ray patterns (see Figure 8): despite the very good alignment of the smectic layers deduced from the narrow first and second order spots on the meridian, the diffuse band in the wide angle region occurring from the lateral packing does not lie on the equator but spreads over the whole azimuthal range.

**Figure 9:**
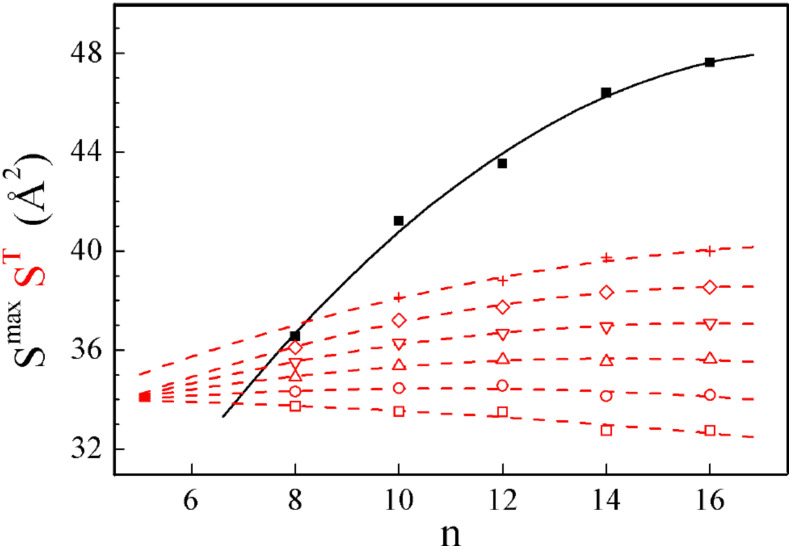
Variations with chain length of the maximum molecular areas close to isotropization S^max^ and of the molecular area isotherms S^T^ in the smectic A phase for the series **1*_n_*** with Br^−^ counter-ion: solid squares: S^max^; open squares: S^60 °C^; open circles: S^80 °C^; open up triangles: S^100 °C^; open down triangles: S^120 °C^; open diamonds: S^140 °C^, crosses S^160 °C^; solid line and dotted lines are parabolic fit of S^max^ and of S^T^, respectively.

Unlike temperature and counter ion size, a chain length increase induces only minor changes of S, the main effect being the evolution toward steeper variations versus *T* (Figure SI-2 in Supporting Information File 1). When plotting together the area isotherms S^T^ and the area close to isotropization S^max^, differences in steepness and gaps clearly set the lower limit for the smectic packing (Figure 9).

Remarkably, the gaps between the extrapolated isotherms seem to vanish close to this low limit, for a chain length corresponding roughly to the segments incorporated in the interface layer, because of the gathering of the ionic sublayers. (On the basis of previous results [32], the calculated molecular area S^0^ and ionic sublayer thickness d_c_^0^ for flat layers are estimated to be 54 Å^2^ and 10 Å, respectively. S and d_c_ values close to the low limit are of about 33 Å^2^ and 17 Å. With the hypothesis that the interface layer goes from d_c_^0^ to (2d_c_−d_c_^0^), the calculation results in chain segments incorporated in the interface layer that contain on average about 7 methylene groups). Thus, this would logically mean that smectic packing is only possible if the tails are long enough to jut out of the diffuse interface with the ionic sublayers and that the tail segments incorporated in the interface do not contribute significantly to the weak dependence of S upon chain length. Beyond the short chain limit, the major influence of chain length concerns the stability of the smectic packing revealed by the variation of S^max^. Thus, increasing chain length causes an important stabilization in the whole explored domain, probably because of the better defined sublayer alternation, extending the smectic packing to larger molecular areas and to more disorganized chains. Nevertheless, increasing chain length also dilutes the ionic sublayers, reducing the correlations between them, and a higher limit should therefore exist for the system, but for much longer tails than the longest grafted here (*n* = 16).

### Conclusion

We have synthesised different series of thermotropic ionic liquid crystals based on the imidazolium cation and containing anions varying with regard to their shapes, sizes and charge localization. As expected, most of the imidazolium compounds show stable mesomorphism, which can be observed at sub-ambient to very high temperature. Both the alkyl chain length and the anion type have a strong influence on the mesomorphic behaviour.

In regard to the different studies realized and more precisely to the dilatometry analysis, the most interesting feature of this imidazolium architecture is its flexibility. This is highlighted, in the smectic A organization, by the ability of the system to compensate the difference between the cross-section of the tails and the ionic lattice area by the gathering of the ionic sublayer and by folding of the tails. This environment-flexible cationic organic backbone offers us, even in presence of large counterions, the opportunity to elaborate ionic liquid crystals. The 1-(4-alkoxybenzyl)-3-methyl-1*H*-imidazol-3-ium cationic moiety is a versatile smectogenic part which could in the near future organise into supramolecular arrangements involving numerous anions with specific functionalities.

## Experimental

### General procedure for imidazolium syntheses

The reactions were performed under Argon atmosphere. 4-(Alkyloxyphenyl)methanol (**A*_n_***) and thionyl bromide were dissolved in dry DCM. TLC monitored the progress of reaction until no more alcohol was observed. The solvent was removed under vacuum and the intermediate 1-bromomethyl-4-alkyloxybenzene was used directly without further purification. This bromo derivative and freshly distilled 1-methylimidazole were stirred in THF during two days. After evaporation to dryness, the residue was purified by flash chromatography on silica gel column (elution DCM/MeOH, MeOH 0% to 9%) followed by flash chromatography on alumina gel column (elution DCM/MeOH, MeOH 3% to 8%) or by a recrystallisation from DCM/Et_2_O.

### General procedure for anion metathesis in water

#### C*_n_*H_2_*_n_*_+1_ with *n* ≤ 10

The lithium, sodium or potassium salt of our desired anion was dissolved in water and added to an aqueous solution of a 1-(4-alkyloxybenzyl)-3-methyl-1*H*-imidazol-3-ium bromide. A slight precipitation of the desired imidazolium occurred immediately. The reaction was stirred for a couple of hours, and dichloromethane was added. The organic layer was washed three times with water, dried on MgSO_4_ and evaporated under reduced pressure.

#### C*_n_*H_2_*_n_*_+1_ with *n* > 10

The lithium, sodium or potassium salt of our desired anion was dissolved in water and added to an aqueous solution of a 1-(4-alkyloxybenzyl)-3-methyl-1*H*-imidazol-3-ium bromide. A precipitation of the desired imidazolium salt occurred immediately. The reaction was stirred for a couple of hours. After centrifugation, the solvent was removed and the crude product was washed twice with water. The product was finally obtained pure by crystallization on a mixture from dichloromethane and diethyl ether.

## Supporting Information

File 1 Product characterization (spectroscopic and analytical data), complementary information about the characterization of the liquid crystalline properties (DSC, X-Ray, dilatometry).
