# Supplementary material for: 1-(4-Alkyloxybenzyl)-3-methyl-1H-imidazol-3-ium organic backbone: A versatile smectogenic moiety
Source: Beilstein J Org Chem. 2009 Nov 6;5:62. doi: 10.3762/bjoc.5.62 (PMC2839789; doi:10.3762/bjoc.5.62)
Supplement: File 1 — Product characterization (spectroscopic and analytical data), complementary information about the characterization of the liquid crystalline properties (DSC, X-Ray, dilatometry). [file Beilstein_J_Org_Chem-05-62-s001.doc]

1-(4-Alkyloxybenzyl)-3-methyl-*1H*-imidazol-3-ium organic backbone: A versatile smectogenic moiety

William Dobbs*, Laurent Douce* and Benoît Heinrich

Institut de Physique et Chimie des Matériaux de Strasbourg, UMR 7504, CNRS-Université de Strasbourg, BP 43, 23 rue du Loess, F-67034 Strasbourg Cedex 2

* Corresponding authors

Solvent and Reagents

The commercial products came from Aldrich, Acros, Fluka or Strem Chemical and were used without further purification, except specific indication in this experimental section.

All the organic solvent were distilled before their use:

- Dichloromethane was distilled on CaH_2_

- Tetrahydrofuran was dried on NaOH before its distillation on Na/benzophenone

The column chromatography were performed on silica gel (Merck 60, 0.04–0.063 μm) and on alumina (aluminium oxyde 90 standard, from Merck)

Analytical Measurements

- **The NMR spectroscopy experiments** were performed with a Bruker Avance300 spectrometer at the resonating frequencies 300.13 MHz for ^1^H NMR spectra and at 75.48 MHz for ^13^C NMR spectra. CDCl_3_ or CD_2_Cl_2_ were used as solvent for the NMR experiments. For internal calibration the residual solvent peak of CDCl_3_ (δ (^1^H) = 7.27 ppm; δ (^13^C)= 77 ppm) and CD_2_Cl_2_ (δ (^1^H) = 5.32 ppm) were used. The peak attribution was listed as below:

- Chemical shift[[1]](#footnote-2) (multiplicity[[2]](#footnote-3), integration, coupling constant[[3]](#footnote-4), attribution)

- **Infrared spectra** were performed with a Perkin–Elmer Spectrum One spectrophotometer equipped with a ATR (Attenuated Transmitted Reflectance) or with a FT-IR Digilab Excalibur FTS 3000 (In this case, we have prepared IR sample as KBr pastille).

- **Elemental Analysis**: The elemental analyses were performed by the elemental analysis department of the University Louis Pasteur or by the central analytical department of the CNRS (Solaize).

- **Polarized optical microscopy studies**: The optical textures of the mesophases were studied with a Leitz polarizing microscope equipped with a Mettler FP80 hot-stage and an FP80 central processor.

- **Thermic measurements:** The transition temperatures and enthalpies were measured by differential scanning calorimetry with a DSC Q1000 from TA Instruments operated at different temperature rates (5 °C·min^−1^ , 2 °C·min^−1^ ) on heating and cooling. The apparatus was calibrated with indium (156.6 °C, 28.4 J·g^−1^ ) and gallium (29.8 °C) as the standards.

The TGA measurements were carried out on a SDTQ 600 apparatus at scanning rate of 10 °C·min^−1^  in air.

- **XRD measurements** were performed on three different lines to obtain complementary data at the small angles between 2 Å to 300 Å. Each of them was equipped with a Cu source (Cu[K_α1_], λ= 1.51418 Å) focalised by a quartz monochromator in the transmission Guinier geometry.

For our compounds, the sample was directly filled in Lindemann capillaries of 1 mm diameter. Our XRD patterns were recorded respectively by curve counter (CPS120 model from INEL) or on image plates (scanned by STORM 820 from Molecular Dynamics with 50 µm resolution).

Analytical Analysis

**1_8_: 1-(4-Octyloxybenzyl)-3-methyl-1*H*-imidazol-3-ium bromide**

**A_8_**: 2 g (8.5 mmol)

Thionyl bromide: 0.78 mL (10 mmol)

Dichloromethane: 20 mL

*N*-Methylimidazole: 0.8 mL (10 mmol)

Tetrahydrofuran: 10 mL

Yield: 2.71 g, 7.14 mmol (84%)

^1^H NMR (300 MHz; CDCl_3_)

δ: 0.87 (t, 3H, ^3^*J* (H,H) = 7.2 Hz, CH_3_ aliphatic chain); 1.28 (m, 8H, CH_2_ aliphatic chain); 1.37–1.42 (m, 2H, CH_2_ aliphatic chain); 1.75 (q, 2H, ^3^*J* (H,H) = 6.8 Hz, O–CH_2_–CH_2_); 3.91 (t, 2H, ^3^*J* (H,H) = 6.6 Hz, OCH_2_); 4.05 (s, 3H, N–CH_3_); 5.48 (s, 2H, Ph–CH_2_–N); 6.86 (d, 2H, ^3^*J* (H,H) = 8.6 Hz, CH phenyl); 7.29 (s, 1H, CH imidazolium); 7.39 (s, 1H, CH imidazolium); 7.41 (d, 2H, ^3^*J* (H,H) = 8.6 Hz, CH phenyl); 10.39 (s, 1H, CH imidazolium).

^13^C NMR (75 MHz; CDCl_3_ {H})

δ: 13.93 (CH_3_ aliphatic chain); 22.47; 25.85; 29.00; 29.05; 29.17; 31.63 (CH_2_ aliphatic chain); 36.62 (N–CH_3_); 52.74 (N–CH_2_–Ph); 68.01 (O–CH_2_); 115.10 (CH phenyl); 121.62; 123.46 (CH imidazolium); 124.60 (C phenyl); 130.49 (CH phenyl); 136.81 (N–CH–N); 159.85 (C phenyl).

IR: ν_max_/cm^−1^  3421 (H_2_O) 3133 (C–H aromatic), 2924 and 2855 (C–H aliphatic), 1513 (C=C aromatic), 1248 (aromatic ether).

Elemental analysis (%) calcd for C_19_H_29_BrN_2_O·1.5 H_2_O: C, 55.9; H, 7.9; N, 6.9; found: C, 55.45; H, 8.00; N, 6.8

**2_8_: 1-(4-Octyloxybenzyl)-3-methyl-1*H*-imidazol-3-ium tetrafluoroborate**

Sodium tetrafluoroborate: 0.22 g (2 mmol)

1-(4-Octyloxybenzyl)-3-methyl-1*H*-imidazol-3-ium bromide: 0.5 g (1.3 mmol)

Water: 10 mL

Yield: 0.47 g, 1.2 mmol (93%)

^1^H NMR (300 MHz; CDCl_3_)

δ: 0.89 (t, 3H, ^3^*J* (H,H) = 7,0 Hz, CH_3_ aliphatic chain); 1.27–1.31 (m, 8H, CH_2_ aliphatic chain); 1.41 (m, 2H, CH_2_ aliphatic chain); 1.76 (q, 2H, ^3^*J* (H,H) = 6,8 Hz, O–CH_2_–CH_2_); 3.90 (t, 2H, ^3^*J* (H,H) = 6,6 Hz, OCH_2_); 3.94 (s, 3H, N–CH_3_); 5.23 (s, 2H, Ph–CH_2_–N); 6.88 (d, 2H, ^3^*J* (H,H) = 8,6 Hz, CH phenyl); 7.17 (m, 1H, CH imidazolium); 7.25 (m, 1H, CH imidazolium); 7.32 (d, 2H, ^3^*J* (H,H) = 8.6 Hz, CH phenyl); 8.83 (s, 1H, CH imidazolium).

^13^C NMR (75 MHz; CDCl_3_ {H})

δ: 14.04 (CH_3_ aliphatic chain); 22.61; 25.99; 29.16; 29.19; 29.32; 31.78 (CH_2_ aliphatic chain); 36.29 (N–CH_3_); 53.07 (N–CH_2_–Ph); 68.17 (O–CH_2_); 115.34 (CH phenyl); 121.64; 123.50 (CH imidazolium); 124.31 (C phenyl); 130.57 (CH phenyl); 136.38 (N–CH–N); 160.10 (C phenyl).

IR: ν_max_/cm^−1^  3161 (C–H aromatic), 2923 and 2854 (C–H aliphatic), 1515 (C=C aromatic), 1249 (aromatic ether), 1036 (BF_4_^−^)

Elemental analysis (%) calcd for C_19_H_29_BF_4_N_2_O·¼ H_2_O: C, 58.1; H, 7.6; N, 7.1; found: C, 58.2; H, 7.6; N, 7.1

**3_8_: 1-(4-Octyloxybenzyl)-3-methyl-1*H*-imidazol-3-ium hexafluorophosphate**

Potassium hexafluorophosphate: 0.239 g (1.3 mmol)

1-(4-Octyloxybenzyl)-3-methyl-1*H*-imidazol-3-ium bromide: 0.5 g (1.3 mmol)

Water: 10 mL

Yield: 0.49 g, 1.1 mmol (84%)

^1^H NMR (300 MHz; CDCl_3_)

δ: 0.89 (t, 3H, ^3^*J* (H,H) = 7,0 Hz, CH_3_ aliphatic chain); 1.26–1.30 (m, 8H, CH_2_ aliphatic chain); 1.40 (m, 2H, CH_2_ aliphatic chain); 1.76 (q, 2H, ^3^*J* (H,H) = 6,8 Hz, O–CH_2_–CH_2_); 3.86 (s, 3H, N–CH_3_); 3.91 (t, 2H, ^3^*J* (H,H) = 6,6 Hz, OCH_2_); 5.18 (s, 2H, Ph–CH_2_–N); 6.88 (d, 2H, ^3^*J* (H,H) = 8,6 Hz, CH phenyl); 7.13 (s, 1H, CH imidazolium); 7.21 (s, 1H, CH imidazolium); 7.29 (d, 2H, ^3^*J* (H,H) = 8.6 Hz, CH phenyl); 8.53 (s, 1H, CH imidazolium).

^13^C NMR (75 MHz; CDCl_3_ {H})

δ: 14.06 (CH_3_ aliphatic chain); 22.62; 25.8; 29.14; 29.19; 29.31; 31.78 (CH_2_ aliphatic chain); 36.20 (N–CH_3_); 53.06 (N–CH_2_–Ph); 68.15 (O–CH_2_); 115.34 (CH phenyl); 121.65; 123.55 (CH imidazolium); 124.99 (C phenyl); 130.57 (CH phenyl); 135.80 (N–CH–N); 160.12 (C phenyl).

IR: ν_max_/cm^−1^  3178 (C–H aromatic), 2918 and 2852 (C–H aliphatic), 1516 (C=C aromatic), 1251 (aromatic ether), 823 (PF_6_^−^)

Elemental analysis (%) calcd for C_19_H_29_F_6_N_2_OP·¾ H_2_O: C, 49.5; H, 6.7; N, 6.1; found: C, 49.6; H, 6.4; N, 6.0

**4_8_: 1-(4-Octyloxybenzyl)-3-methyl-1*H*-imidazol-3-ium thiocyanate**

Sodium thiocyanate: 0.065 g (0.66 mmol)

1-(4-Octyloxybenzyl)-3-methyl-1*H*-imidazol-3-ium bromide: 0.25 g (0.65 mmol)

Yield: 0,187 g, 0.53 mmol (80%)

^1^H NMR (300 MHz; CD_2_Cl_2_)

δ: 0.88 (t, 3H, ^3^*J* (H,H) = 7.0 Hz, CH_3_ aliphatic chain); 1.29 (m, 8H, CH_2_ aliphatic chain); 1.43 (m, 2H, CH_2_ aliphatic chain); 1.76 (q, 2H, ^3^*J* (H,H) = 6.6 Hz, O–CH_2_–CH_2_); 3.94 (t, 2H, ^3^*J* (H,H) = 6.6 Hz, OCH_2_); 3.99 (s, 3H, N–CH_3_); 5.38 (s, 2H, Ph–CH_2_–N); 6.91 (d, 2H, ^3^*J* (H,H) = 8,6 Hz, CH phenyl); 7.29 (s, 1H, CH imidazolium); 7.33 (s, 1H, CH imidazolium); 7.39 (d, 2H, ^3^*J* (H,H) = 8.6 Hz, CH phenyl); 9.33 (s, 1H, CH imidazolium).

^13^C NMR (75 MHz; CD_2_Cl_2_ {H})

δ: 14.20 (CH_3_ aliphatic chain); 22.99; 26.33; 29.53; 29.57; 29.68; 32.16 (CH_2_ aliphatic chain); 37.02 (N–CH_3_); 53.64 (N–CH_2_–Ph); 68.63 (O–CH_2_); 115.62 (CH phenyl); 122.25; 123.96 (CH imidazolium); 124.86 (C phenyl); 130.99 (CH phenyl); 137.17 (N–CH–N); 131.78 (SCN); 160.55 (C phenyl).

IR: ν_max_/cm^−1^  3350 (H_2_O), 3095 (C–H aromatic), 2921 and 2854 (C–H aliphatic), 2052 (SCN^−^) 1514 (C=C aromatic), 1248 (aromatic ether)

Elemental analysis (%) calcd for C_20_H_29_N_3_OS·H_2_O: C, 63.6; H, 8.3; N, 11.1; found: C, 63.3; H, 8.25; N, 11.0

**5_8_: 1-(4-Octyloxybenzyl)-3-methyl-1*H*-imidazol-3-ium trifluoromethanesulfonate**

Sodium trifluoromethanesulfonate: 0.114 g (0.66 mmol)

1-(4-Octyloxybenzyl)-3-methyl-1*H*-imidazol-3-ium bromide: 0.25 g (0.66 mmol)

Water: 15 mL

Yield: 0.248 g, 0,55 mmol (83%)

^1^H NMR (300 MHz; CDCl_3_)

δ: 0.89 (t, 3 H, ^3^*J* (H,H) = 6.8 Hz, CH_3_ aliphatic chain); 1.31 (m, 8H, CH_2_ aliphatic chain); 1.44 (m, 2H, CH_2_ aliphatic chain); 1.77 (q, 2 H, ^3^*J* (H,H) = 6.6 Hz, O–CH_2_–CH_2_); 3.94 (t, 2 H, ^3^*J* (H,H) = 6.6 Hz, OCH_2_); 3.95 (s, 3 H, CH_3_–N); 5.28 (s, 2 H, N–CH_2_–Ph); 6.90 (d, 2 H, ^3^*J* (H,H) = 8.6 Hz, CH phenyl); 7.17 (m, 1 H, CH imidazolium); 7.25 (m, 1 H, CH imidazolium); 7.33 (d, 2 H, ^3^*J* (H,H) = 8.6 Hz, CH phenyl); 9.20 (s, 1 H, N–CH–N).

^13^C NMR (75 MHz; CDCl_3_ {H})

δ: 14.05 (CH_3_ aliphatic chain); 22.63; 25.99; 29.14; 29.20; 29.32; 31.78 (CH_2_ aliphatic chain); 36.46 (N–CH_3_); 53.23 (N–CH_2_–Ph); 68.21 (O–CH_2_); 115.41 (CH phenyl); 119.12 (q, *J (*C–F)= 317.5 Hz, CF_3_); 121.62; 123.39 (CH imidazolium); 124.10 (C phenyl); 130.57 (CH phenyl); 136.89 (N–CH–N); 160.09 (C phenyl).

IR: ν_max_/cm^−1^  3122 (C–H aromatic), 2921 and 2851 (C–H aliphatic), 1516 (C=C aromatic), 1249 and 1029 (CF_3_SO_3_^−^).

Elemental analysis (%) calcd for C_20_H_29_F_3_N_2_O_4_S·¼ H_2_O: C, 52.8; H, 6.5; N, 6.2; found: C, 52.7; H, 6.6; N, 6.1

**6_8_: 1-(4-Octyloxybenzyl)-3-methyl-1*H*-imidazol-3-ium bis[(trifluoromethyl)sulfonyl]amide**

Lithium bis[(trifluoromethyl)sulfonyl]amide: 0.207 g (0.72 mmol)

1-(4-Octyloxybenzyl)-3-methyl-1*H*-imidazol-3-ium bromide: 0.25 g (0.65 mmol)

Yield: 0.28 g, 0.48 mmol (74%)

^1^H NMR (300 MHz; CDCl_3_)

δ: 0.90 (t, 3H, ^3^*J* (H,H) = 7,0 Hz, CH_3_ aliphatic chain); 1.31 (m, 8H, CH_2_ aliphatic chain); 1.45 (m, 2H, CH_2_ aliphatic chain); 1.79 (q, 2H, ^3^*J* (H,H) = 6,8 Hz, O–CH_2_–CH_2_); 3.95 (s, 3H, N–CH_3_); 3.96 (t, 2H, ^3^*J* (H,H) = 6,6 Hz, OCH_2_); 5.26 (s, 2H, Ph–CH_2_–N); 6.93 (d, 2H, ^3^*J* (H,H) = 8,6 Hz, CH phenyl); 7.14 (s, 1H, CH imidazolium); 7.20 (s, 1H, CH imidazolium); 7.30 (d, 2H, ^3^*J* (H,H) = 8.6 Hz, CH phenyl); 8.82 (s, 1H, CH imidazolium).

^13^C NMR (75 MHz; CDCl_3_ {H})

δ: 14.05 (CH_3_ aliphatic chain); 22.63; 25.99; 29.14; 29.20; 29.31; 31.79 (CH_2_ aliphatic chain); 36.54 (N–CH_3_); 53.46 (N–CH_2_–Ph); 68.26 (O–CH_2_); 115.55 (CH phenyl); 119.83 (quadruplet, *J* (C,F)= 319.1 Hz, CF3); 121.77; 123.42 (CH imidazolium); 123.51 (C phenyl); 130.59 (CH phenyl); 136.23 (N–CH–N); 160.41 (C phenyl).

IR: ν_max_/cm^−1^  3154 (C–H aromatic), 2929 and 2858 (C–H aliphatic), 1516 (C=C aromatic), 1349 and 1178 ([CF_3_SO_2_]_2_N^−^)

Elemental analysis (%) calcd for C_21_H_29_F_6_N_3_O_5_S_2_·½ H_2_O: C, 42.7; H, 5.1; N, 7.1; found: C, 42.7; H, 4.9; N, 7.0

**1_10_: 1-(4-Decyloxybenzyl)-3-methyl-1*H*-imidazol-3-ium bromide**

**A_10_**: 3 g (11.3 mmol)

Thionyl bromide: 1 mL (13 mmol)

Dichloromethane: 20 mL

*N*-Methylimidazole: 1 mL (12.5 mmol)

Tetrahydrofuran: 10 mL

Yield: 3.9 g, 9,61 mmol (85%)

^1^H NMR (300 MHz; CDCl_3_)

δ: 0.88 (t, 3H, ^3^*J* (H,H) = 7.2 Hz, CH_3_ aliphatic chain); 1.27 (br s, 12H, CH_2_ aliphatic chain); 1.41 (m, 2H, CH_2_ aliphatic chain); 1.77 (q, 2H, ^3^*J* (H,H) = 6.8 Hz, O–CH_2_–CH_2_); 3.93 (t, 2H, ^3^*J* (H,H) = 6.6 Hz, OCH_2_); 4.07 (s, 3H, N–CH_3_); 5.48 (s, 2H, Ph–CH_2_–N); 6.89 (d, 2H, ^3^*J* (H,H) = 8.6 Hz, CH phenyl); 7.18 (s, 1H, CH imidazolium); 7.26 (s, 1H, CH imidazolium); 7.41 (d, 2H, ^3^*J* (H,H) = 8.6 Hz, CH phenyl); 10.60 (s, 1H, CH imidazolium).

^13^C NMR (75 MHz; CDCl_3_ {H})

δ: 14.07 (CH_3_ aliphatic chain); 22.64; 25.98; 29.13; 29.27; 29.34; 29.53; 31.86 (CH_2_ aliphatic chain); 36.75 (N–CH_3_); 53.14 (N–CH_2_–Ph); 68.18 (O–CH_2_); 115.34 (CH phenyl); 121.33; 122.97 (CH imidazolium); 124.30 (C phenyl); 130.63 (CH phenyl); 137.76 (N–CH–N); 160.14 (C phenyl).

IR: ν_max_/cm^−1^  3408 (H_2_O) 3133 (C–H aromatic), 2921 and 2853 (C–H aliphatic), 1512 (C=C aromatic), 1246 (aromatic ether).

Elemental analysis (%) calcd for C_21_H_33_BrN_2_O·H_2_O: C, 59.0; H, 8.25; N, 6.55; found: C, 59.2; H, 8.1; N, 6.5

**2_10_: 1-(4-Decyloxybenzyl)-3-methyl-1*H*-imidazol-3-ium tetrafluoroborate**

Potassium tetrafluoroborate: 0.568 g (5.2 mmol)

1-(4-Decyloxybenzyl)-3-methyl-1*H*-imidazol-3-ium bromide: 2 g (4.9 mmol)

Yield: 1.9 g, 4.84 mmol (93%)

^1^H NMR (300 MHz; CDCl_3_)

δ: 0.88 (t, 3H, ^3^*J* (H,H) = 6.8 Hz, CH_3_ aliphatic chain); 1.28 (br s, 12H, CH_2_ aliphatic chain); 1.42 (m, 2H, CH_2_ aliphatic chain); 1.77 (q, 2H, ^3^*J* (H,H) = 6.8 Hz, O–CH_2_–CH_2_); 3.91 (s, 3H, N–CH_3_); 3.92 (t, 2H, ^3^*J* (H,H) = 6.6 Hz, OCH_2_); 5.24 (s, 2H, Ph–CH_2_–N); 6.88 (d, 2H, ^3^*J* (H,H) = 8.6 Hz, CH phenyl); 7.17 (m, 1H, CH imidazolium); 7.25 (m, 1H, CH imidazolium); 7.30 (d, 2H, ^3^*J* (H,H) = 8.6 Hz, CH phenyl); 8.86 (s, 1H, CH imidazolium).

^13^C NMR (75 MHz; CDCl_3_ {H})

δ:14.07 (CH_3_ aliphatic chain); 22.64; 25.99; 29.16; 29.28; 29.37; 29.53; 31.86 (CH_2_ aliphatic chain); 36.31 (N–CH_3_); 53.08 (N–CH_2_–Ph); 68.18 (O–CH_2_); 115.34 (CH phenyl); 121.62; 123.49 (CH imidazolium); 124.30 (C phenyl); 130.59 (CH phenyl); 136.41 (N–CH–N); 160.11 (C phenyl).

IR: ν_max_/cm^−1^  3157 (C–H aromatic), 2920 and 2852 (C–H aliphatic), 1515 (C=C aromatic), 1252 (aromatic ether), 1049 (BF_4_^−^)

Elemental analysis (%) calcd for C_21_H_33_BF_4_N_2_O: C, 60.6; H, 8.0; N, 6.7; found: C, 60.5; H, 7.9; N, 6.7

**3_10_: 1-(4-Decyloxybenzyl)-3-methyl-1*H*-imidazol-3-ium hexafluorophosphate**

Potassium hexafluorophosphate: 0.341 g (1.9 mmol)

1-(4-Decyloxybenzyl)-3-methyl-1*H*-imidazol-3-ium bromide: 0.5 g (1.2 mmol)

Yield: 0.48 g, 0.96 mmol (80%)

^1^H NMR (300 MHz; CDCl_3_)

δ: 0.88 (t, 3H, ^3^*J* (H,H) = 6.8 Hz, CH_3_ aliphatic chain); 1.28 (br s, 12H, CH_2_ aliphatic chain); 1.38–1.43 (m, 2H, CH_2_ aliphatic chain); 1.77 (q, 2H, ^3^*J* (H,H) = 6,8 Hz, O–CH_2_–CH_2_); 3.92 (s, 3H, N–CH_3_); 3.94 (t, 2H, ^3^*J* (H,H) = 6,6 Hz, OCH_2_); 5.23 (s, 2H, Ph–CH_2_–N); 6.92 (d, 2H, ^3^*J* (H,H) = 8,6 Hz, CH phenyl); 7.11 (s, 1H, CH imidazolium); 7.18 (s, 1H, CH imidazolium); 7.30 (d, 2H, ^3^*J* (H,H) = 8.6 Hz, CH phenyl); 8.69 (s, 1H, CH imidazolium).

^13^C NMR (75 MHz; CDCl_3_ {H})

δ: 14.07 (CH_3_ aliphatic chain); 22.64; 25.98; 29.15; 29.29; 29.38; 29.54; 31.86 (CH_2_ aliphatic chain); 36.21 (N–CH_3_); 53.07 (N–CH_2_–Ph); 68.16 (O–CH_2_); 115.34 (CH phenyl); 121.65; 123.53 (CH imidazolium); 123.99 (C phenyl); 130.57 (CH phenyl); 135.84 (N–CH–N); 160.12 (C phenyl).

IR: ν_max_/cm^−1^  3179 (C–H aromatic), 2918 and 2851 (C–H aliphatic), 1517 (C=C aromatic), 1252 (aromatic ether), 822 (PF_6_^−^).

Elemental analysis (%) calcd for C_21_H_33_F_6_N_2_OP: C, 53.2; H, 7.0; N, 5.9; found: C, 53.5; H, 6.9; N, 5.8

**4_10_: 1-(4-Decyloxybenzyl)-3-methyl-1*H*-imidazol-3-ium thiocyanate**

Potassium thiocyanate: 0.25 g (2.56 mmol)

1-(4-Decyloxybenzyl)-3-methyl-1*H*-imidazol-3-ium bromide: 0.5 g (1.28 mmol)

Yield: 0.4 g, 1.02 mmol(80%)

^1^H NMR (300 MHz; CDCl_3_)

δ: 0.88 (t, 3H, ^3^*J* (H,H) = 6.8 Hz, CH_3_ aliphatic chain); 1.27 (br s, 12H, CH_2_ aliphatic chain); 1.42 (m, 2H, CH_2_ aliphatic chain); 1.76 (q, 2H, ^3^*J* (H,H) = 6,8 Hz, O–CH_2_–CH_2_); 3.93 (t, 2H, ^3^*J* (H,H) = 6,6 Hz, OCH_2_); 4.06 (s, 3H, N–CH_3_); 5.42 (s, 2H, Ph–CH_2_–N); 6.91 (d, 2H, ^3^*J* (H,H) = 8,7 Hz, CH phenyl); 7.28 (m, 1H, CH imidazolium); 7.36 (m, 1H, CH imidazolium); 7.39(d, 2H, ^3^*J* (H,H) = 8.7 Hz, CH phenyl); 9.45 (s, 1H, CH imidazolium).

^13^C NMR (75 MHz; CDCl_3_ {H})

δ:14.05 (CH_3_ aliphatic chain); 22.61; 25.97; 29.13; 29.25; 29.33; 29.50; 29.51; 31.83 (CH_2_ aliphatic chain); 36.79 (N–CH_3_); 53.41 (N–CH_2_–Ph); 68.19 (O–CH_2_); 115.40 (CH phenyl); 121.80; 123.50 (CH imidazolium); 124.15 (C phenyl); 130.70 (CH phenyl); 131.67 (SCN); 136.89 (N–CH–N); 160.17 (C phenyl).

IR: ν_max_/cm^−1^  3290 (H_2_O) 3147 (C–H aromatic), 2917 and 2850 (C–H aliphatic), 2063 (SCN^−^) 1514 (C=C aromatic), 1249 (aromatic ether).

Elemental analysis (%) calcd for C_22_H_33_N_3_OS·H_2_O: C, 65.15; H, 8.7; N, 10.4; found: C, 65.3; H, 8.75; N, 10.2

**5_10_: 1-(4-Decyloxybenzyl)-3-methyl-1*H*-imidazol-3-ium trifluoromethanesulfonate**

Sodium trifluoromethanesulfonate: 0.42 g (2.44 mmol)

1-(4-Decyloxybenzyl)-3-methyl-1*H*-imidazol-3-ium bromide: 0.5 g (1.22 mmol)

Water: 20 mL

Yield: 0.42 g, 0,86 mmol (72%)

^1^H NMR (300 MHz; CDCl_3_)

δ: 0.89 (t, 3 H, ^3^*J* (H,H) = 6.8 Hz, CH_3_ aliphatic chain); 1.27 (br s, 12 H, CH_2_ aliphatic chain); 1.35–1.48 (m, 2 H, CH_2_ aliphatic chain); 1.78 (q, 2 H, ^3^*J* (H,H) = 6.6 Hz, O–CH_2_–CH_2_); 3.94 (t, 2 H, ^3^*J* (H,H) = 6.6 Hz, OCH_2_); 3.96 (s, 3 H, CH_3_–N); 5.28 (s, 2 H, N–CH_2_–Ph); 6.91 (d, 2 H, ^3^*J* (H,H) = 8.7 Hz, CH phenyl); 7.16 (m, 1 H, CH imidazolium); 7.23 (m, 1 H, CH imidazolium); 7.31 (d, 2 H, ^3^*J* (H,H) = 8.7 Hz, CH phenyl); 9.22 (s, 1 H, N–CH–N).

^13^C NMR (75 MHz; CDCl_3_ {H})

δ:14.07 (CH_3_ aliphatic chain); 22.65; 25.99; 29.15; 29.29; 29.36; 29.54; 31.87 (CH_2_ aliphatic chain); 36.49 (N–CH_3_); 53.29 (N–CH_2_–Ph); 68.22 (O–CH_2_); 115.43 (CH phenyl); 120.70 (q, *J (*C–F)= 318 Hz, CF_3_); 121.57; 123.30 (CH imidazolium); 124.02 (C phenyl); 130.59 (CH phenyl); 137.01 (N–CH–N); 160.25 (C phenyl).

IR: ν_max_/cm^−1^  3157 (C–H aromatic), 2919 and 2849 (C–H aliphatic), 1517 (C=C aromatic), 1247 and 1030 (CF_3_SO_3_^−^).

Elemental analysis (%) calcd for C_22_H_33_F_3_N_2_O_4_S: C, 55.2; H, 6.95; N, 5.85; found: C, 55.2; H, 6.8; N, 5.8

**6_10_: 1-(4-Decyloxybenzyl)-3-methyl-1*H*-imidazol-3-ium bis[(trifluoromethyl)sulfonyl]amide**

Lithium bis[(trifluoromethyl)sulfonyl]amide: 0.193 g (0.67 mmol)

1-(4-Decyloxybenzyl)-3-methyl-1*H*-imidazol-3-ium bromide: 0.25 g (0.61 mmol)

Yield: 0.28 g, 0.46 mmol (76%)

^1^H NMR (300 MHz; CDCl_3_)

δ: 0.89 (t, 3H, ^3^*J* (H,H) = 6.8 Hz, CH_3_ aliphatic chain); 1.28 (br s, 12H, CH_2_ aliphatic chain); 1.42 (m, 2H, CH_2_ aliphatic chain); 1.79 (q, 2H, ^3^*J* (H,H) = 6,8 Hz, O–CH_2_–CH_2_); 3.94 (s, 3H, N–CH_3_); 3.95 (t, 2H, ^3^*J* (H,H) = 6,6 Hz, OCH_2_); 5.26 (s, 2H, Ph–CH_2_–N); 6.92 (d, 2H, ^3^*J* (H,H) = 8,6 Hz, CH phenyl); 7.14 (s, 1H, CH imidazolium); 7.21 (s, 1H, CH imidazolium); 7.30 (d, 2H, ^3^*J* (H,H) = 8.6 Hz, CH phenyl); 8.82 (s, 1H, CH imidazolium).

^13^C NMR (75 MHz; CDCl_3_ {H})

δ: 14.07 (CH_3_ aliphatic chain); 22.66; 25.99; 29.15; 29.30; 29.36; 29.54; 31.88 (CH_2_ aliphatic chain); 36.53 (N–CH_3_); 53.45 (N–CH_2_–Ph); 68.26 (O–CH_2_); 115.53 (CH phenyl); 120.15 (quadruplet, *J* (C,F)= 319.10 Hz, CF_3_); 121.77; 123.45 (CH imidazolium); 123.52 (C phenyl); 130.59 (CH phenyl); 136.20 (N–CH–N); 160.40 (C phenyl).

IR: ν_max_/cm^−1^  3157 (C–H aromatic), 2919 and 2849 (C–H aliphatic), 1517 (C=C aromatic), 1352 and 1181 ([CF_3_SO_2_]_2_N^−^)

Elemental analysis (%) calcd for C_23_H_33_F_6_N_3_O_5_S_2_: C, 45.3; H, 5.5; N, 6.9; found: C, 45.1; H, 5.6; N, 6.8

**1_12_: 1-(4-Dodecyloxybenzyl)-3-methyl-1*H*-imidazol-3-ium bromide**

**A_12_**: 8.2 g (28 mmol)

Thionyl bromide: 2.25 mL (28 mmol)

Dichloromethane: 80 mL

*N*-Methylimidazole: 2.3 mL (28 mmol)

Tetrahydrofuran: 80 mL

Yield: 10.8 g, 24,6 mmol (88%)

^1^H NMR (300 MHz; CDCl_3_)

δ: 0.88 (t, 3 H, ^3^*J* (H,H) = 6.9 Hz, CH_3_ aliphatic chain); 1.27 (br s, 16 H, CH_2_ aliphatic chain); 1.44 (m, 2 H, CH_2_ aliphatic chain); 1.78 (q, 2 H, ^3^*J* (H,H) = 6.9 Hz, O–CH_2_–CH_2_); 3.95 (t, 2 H, ^3^*J* (H,H) = 6.5 Hz, OCH_2_); 4.08 (s, 3 H, CH_3_–N); 5.47 (s, 2 H, N–CH_2_–Ph); 6.91 (d, 2 H, ^3^*J* (H,H) = 8.6 Hz, CH phenyl); 7.09 (m, 1 H, CH imidazolium); 7.13 (m, 1 H, CH imidazolium); 7.40 (d, 2 H, ^3^*J* (H,H) = 8.6 Hz, CH phenyl); 10.89 (s, 1 H, N–CH–N).

^13^C NMR (75 MHz; CDCl_3_ {H})

δ: 13.86 (CH_3_ aliphatic chain); 22.42; 25.76; 28.92; 29.08; 29.15; 29.31; 29.34; 29.36; 29.40; 31.64 (CH_2_ aliphatic chain); 36.52 (N–CH_3_); 52.56 (N–CH_2_–Ph); 67.90 (O–CH_2_); 114.94 (CH phenyl); 121.58; 123.47 (CH imidazolium); 124.61 (C phenyl); 130.39 (CH phenyl); 136.54 (N–CH–N); 159.71 (C phenyl).

IR: ν_max_/cm^−1^  3133 (C–H aromatic), 2919 and 2850 (C–H aliphatic), 1513 (C=C aromatic), 1248 (aromatic ether).

Elemental analysis (%) calcd for C_23_H_37_BrN_2_O: C, 63.15; H, 8.5; N, 6.4; found: C, 62.7; H, 8.55; N, 6.45

**2_12_: 1-(4-Dodecyloxybenzyl)-3-methyl-1*H*-imidazol-3-ium tetrafluoroborate**

Sodium tetrafluoroborate: 0.064 g (0.57 mmol)

1-(4-Dodecyloxybenzyl)-3-methyl-1*H*-imidazol-3-ium bromide: 0.25 g (0.57 mmol)

Yield: 0.25 g, 0,56 mmol (98%)

^1^H NMR (300 MHz; CDCl_3_)

δ: 0.89 (t, 3 H, ^3^*J* (H,H) = 6.8 Hz, CH_3_ aliphatic chain); 1.27 (br s, 16 H, CH_2_ aliphatic chain); 1.42 (m, 2 H, CH_2_ aliphatic chain); 1.76 (q, 2 H, ^3^*J* (H,H) = 6.8 Hz, O–CH_2_–CH_2_); 3.94 (t, 2 H, ^3^*J* (H,H) = 6.5 Hz, OCH_2_); 3.98 (s, 3 H, CH_3_–N); 5.29 (s, 2 H, N–CH_2_–Ph); 6.91 (d, 2 H, ^3^*J* (H,H) = 8.6 Hz, CH phenyl); 7.09 (m, 1 H, CH imidazolium); 7.16 (m, 1 H, CH imidazolium); 7.33 (d, 2 H, ^3^*J* (H,H) = 8.6 Hz, CH phenyl); 9.1 (s, 1 H, N–CH–N).

^13^C NMR (75 MHz; CDCl_3_ {H})

δ: 14.10 (CH_3_ aliphatic chain); 22.67; 26.00; 29.16; 29.33; 29.39; 29.57; 29.59; 29.62; 29.65; 31.90 (CH_2_ aliphatic chain); 36.37 (N–CH_3_); 53.15 (N–CH_2_–Ph); 67.18 (O–CH_2_); 115.35 (CH phenyl); 121.57; 123.38 (CH imidazolium); 124.17 (C phenyl); 130.60 (CH phenyl); 136.56 (N–CH–N); 160.13 (C phenyl).

IR: ν_max_/cm^−1^  3154 (C–H aromatic), 2919 and 2848 (C–H aliphatic), 1515 (C=C aromatic), 1251 (aromatic ether), 1036 (BF_4_^−^)

Elemental analysis (%) calcd for C_23_H_37_BF_4_N_2_O·½ H_2_O: C, 60.9; H, 8.45; N, 6.2; found: 60.6; H, 8.25; N, 6.1

**3_12_: 1-(4-Dodecyloxybenzyl)-3-methyl-1*H*-imidazol-3-ium hexafluorophosphate**

Potassium hexafluorophosphate: 0.105 g

1-(4-Dodecyloxybenzyl)-3-methyl-1*H*-imidazol-3-ium bromide: 0.25 g (0.57 mmol)

Yield: 0.285 g, 0,56 mmol (99%)

^1^H NMR (300 MHz; CDCl_3_)

δ: 0.89 (t, 3 H, ^3^*J* (H,H) = 6.9 Hz, CH_3_ aliphatic chain); 1.27 (br s, 16 H, CH_2_ aliphatic chain); 1.44 (m, 2 H, CH_2_ aliphatic chain); 1.77 (q, 2 H, ^3^*J* (H,H) = 6.9 Hz, O–CH_2_–CH_2_); 3.89 (s, 3 H, CH_3_–N); 3.93 (t, 2 H, ^3^*J* (H,H) = 6.5 Hz, OCH_2_); 5.20 (s, 2 H, N–CH_2_–Ph); 6.90 (d, 2 H, ^3^*J* (H,H) = 8.6 Hz, CH phenyl); 7.11 (m, 1 H, CH imidazolium); 7.20 (m, 1 H, CH imidazolium); 7.30 (d, 2 H, ^3^*J* (H,H) = 8.6 Hz, CH phenyl); 8.58 (s, 1 H, N–CH–N).

^13^C NMR (75 MHz; CDCl_3_ {H})

δ: 14.09 (CH_3_ aliphatic chain); 22.66; 26.00; 29.17; 29.33; 29.40; 29.57; 29.59; 29.62; 29.65; 31.89 (CH_2_ aliphatic chain); 36.21 (N–CH_3_); 53.09 (N–CH_2_–Ph); 68.17 (O–CH_2_); 115.36 (CH phenyl); 121.65; 123.54 (CH imidazolium); 123.96 (C phenyl); 130.58 (CH phenyl); 135.82 (N–CH–N); 160.14 (C phenyl).

IR: ν_max_/cm^−1^  3178 (C–H aromatic), 2916 and 2850 (C–H aliphatic), 1251 (aromatic ether), 823 (PF_6_^(–)^)

Elemental analysis (%) calcd for C_23_H_37_F_6_N_2_OP: C, 55,0; H, 7.4; N, 5.6; found: 54.8; H, 7.0; N, 5.6

**4_12_: 1-(4-Dodecyloxybenzyl)-3-methyl-1*H*-imidazol-3-ium thiocyanate**

Potassium thiocyanate: 0.055 g (0.57 mmol)

1-(4-Dodecyloxybenzyl)-3-methyl-1*H*-imidazol-3-ium bromide: 0.25 g (0.57 mmol)

Water: 20 mL

Yield: 0.226 g, 0.54 mmol (95%)

^1^H NMR (300 MHz; CDCl_3_)

δ: 0.82 (t, 3 H, ^3^*J* (H,H) = 6.8 Hz, CH_3_ aliphatic chain); 1.20 (br s, 16 H, CH_2_ aliphatic chain); 1.35 (m, 2 H, CH_2_ aliphatic chain); 1.71 (q, 2 H, ^3^*J* (H,H) = 6.8 Hz, O–CH_2_–CH_2_); 3.88 (t, 2 H, ^3^*J* (H,H) = 6.5, OCH_2_); 4.02 (s, 3 H, CH_3_–N); 5.36 (s, 2 H, N–CH_2_–Ph); 6.86 (d, 2 H, ^3^*J* (H,H) = 8.6 Hz, CH phenyl); 7.08 (m, 1 H, CH imidazolium); 7.13 (m, 1 H, CH imidazolium); 7.31 (d, 2 H, ^3^*J* (H,H) = 8.6 Hz, CH phenyl); 9.63 (s, 1 H, N–CH–N).

^13^C NMR (75 MHz; CDCl_3_ {H})

δ: 14.05 (CH_3_ aliphatic chain); 22.60; 25.94; 29.10; 29.26; 29.33; 29.50; 29.52; 29.55; 29.58; 31.83 (CH_2_ aliphatic chain); 36.72 (N–CH_3_); 52.26 (N–CH_2_–Ph); 68.12 (O–CH_2_); 115.27 (CH phenyl); 121.80; 123.54 (CH imidazolium); 124.22 (C phenyl); 130.66 (CH phenyl); 131.66 SCN); 136.64 (N–CH–N); 160.01 (C phenyl).

IR: ν_max_/cm^−1^  3147 (C–H aromatic); 2916 and 2849 (C–H aliphatic); 2064 (SCN), 1514 (C=C aromatic); 1249 (aromatic ether)

Elemental analysis (%) calcd for C_24_H_37_N_3_OS·H_2_O: C, 66.5; H, 9.1; N, 9.7; found: 66.3; H, 9.0; N, 9.6

**5_12_: 1-(4-Dodecyloxybenzyl)-3-methyl-1*H*-imidazol-3-ium trifluoromethanesulfonate**

Sodium trifluoromethanesulfonate: 0.099 g (0,57 mmol)

1-(4-Dodecyloxybenzyl)-3-methyl-1*H*-imidazol-3-ium bromide: 0.25 g (0,57 mmol)

Water: 60 mL

Yield: 0.26 g, 0.51 mmol (90%)

^1^H NMR (300 MHz; CDCl_3_)

δ: 0.89 (t, 3 H, ^3^*J* (H,H) = 6.8 Hz, CH_3_ aliphatic chain); 1.27 (br s, 16 H, CH_2_ aliphatic chain); 1.35–1.48 (m, 2 H, CH_2_ aliphatic chain); 1.78 (q, 2 H, ^3^*J* (H,H) = 6.6 Hz, O–CH_2_–CH_2_); 3.95 (t, 2 H, ^3^*J* (H,H) = 6.6 Hz, OCH_2_); 3.99 (s, 3 H, CH_3_–N); 5.31 (s, 2 H, N–CH_2_–Ph); 6.92 (d, 2 H, ^3^*J* (H,H) = 8.6 Hz, CH phenyl); 7.10 (m, 1 H, CH imidazolium); 7.16 (m, 1 H, CH imidazolium); 7.32 (d, 2 H, ^3^*J* (H,H) = 8.6 Hz, CH phenyl); 9.40 (s, 1 H, N–CH–N).

^13^C NMR (75 MHz; CDCl_3_ {H})

δ:14.02 (CH_3_ aliphatic chain); 22.60; 25.94; 29.10; 29.26; 29.26; 29.32; 29.50; 29.53; 29.58; 31.83 (CH_2_ aliphatic chain); 36.26 (N–CH_3_); 52.96 (N–CH_2_–Ph); 68.10(O–CH_2_); 115.23 (CH phenyl); 120.6 (q, *J (*C–F)= 320 Hz, CF_3_); 121.76; 123.61 (CH imidazolium); 124.31 (C phenyl); 130.46 (CH phenyl); 136.34 (N–CH–N); 160.02 (C phenyl).

IR: ν_max_/cm^−1^  3122 (C–H aromatic), 2918 and 2850 (C–H aliphatic), 1516 (C=C aromatic), 1260 and 1029 (CF_3_SO_3_^−^).

Elemental analysis (%) calcd for C_24_H_37_F_3_N_2_O_4_S: C, 56.9; H, 7.4; N, 5.5; found: C, 56.8; H, 7.4; N, 5.6

**6_12_: 1-(4-Dodecyloxybenzyl)-3-methyl-1*H*-imidazol-3-ium bis[(trifluoromethyl)sulfonyl]amide**

Lithium bis[trifluoromethyl)sulfonyl]amide: 0.164 g (0.57 mmol)

1-(4-Dodecyloxybenzyl)-3-methyl-1*H*-imidazol-3-ium bromide: 0.25 g (0.57 mmol)

Water: 20 mL

Yield: 0.363 g, 0.53 mmol (93%)

^1^H NMR (300 MHz; CDCl_3_)

δ: 0.89 (t, 3 H, ^3^*J* (H,H) = 6.8 Hz, CH_3_ aliphatic chain); 1.27 (br s, 16 H, CH_2_ aliphatic chain); 1.44 (m, 2 H, CH_2_ aliphatic chain); 1.79 (m, 2 H, O–CH_2_–CH_2_); 3.96 (t, 2 H, ^3^*J* (H,H) = 6.5 Hz, OCH_2_); 3.99 (s, 3 H, CH_3_–N); 5.28 (s, 2 H, N–CH_2_–Ph); 6.94 (d, 2 H, ^3^*J* (H,H) = 8.6 Hz, CH phenyl); 7.12 (m, 1 H, CH imidazolium); 7.16 (m, 1 H, CH imidazolium); 7.31 (d, 2 H, ^3^*J* (H,H) = 8.6 Hz, CH phenyl); 8.93 (s, 1 H, N–CH–N).

^13^C NMR (75 MHz; CDCl_3_ {H})

δ: 14.04 (CH_3_ aliphatic chain); 22.62; 25.95; 29.12; 29.29; 29.34; 29.52; 29.55; 29.58; 29.60; 31.86 (CH_2_ aliphatic chain); 36.27 (N–CH_3_); 53.15 (N–CH_2_–Ph); 68.15 (O–CH_2_); 115.36 (CH phenyl); 119.76 (quadruplet, *J* (C,F)= 321.2 Hz, CF_3_); 121.89; 123.70 (CH imidazolium); 123.79 (C phenyl); 130.48 (CH phenyl); 135.63 (N–CH–N); 160.20 (C phenyl).

IR: ν_max_/cm^−1^  3156 (C–H aromatic), 2918 and 2848 (C–H aliphatic), 1517 (C=C aromatic), 1353 and 1180 ([CF_3_SO_2_]_2_N)

Elemental analysis (%) calcd for C_25_H_37_F_6_N_3_O_3_S_2_: C, 47.1; H, 5.85; N, 6.6; found: 47.15; H, 5.90; N, 6.6

**1_14_: 1-(4-Tetradecyloxybenzyl)-3-methyl-1*H*-imidazol-3-ium bromide**

**A_14_**: 9.6 g (30 mmol)

Thionyl bromide: 2.3 ml (30 mmol)

Dichloromethane: 50 mL

*N*-Methylimidazole: 3.6 mL (45 mmol)

Tetrahydrofuran: 50 mL

Yield: 12.3 g, 26,4 mmol (88%)

^1^H NMR (300 MHz; CDCl_3_)

δ: 0.88 (t, 3 H, ^3^*J* (H,H) = 6.9 Hz, CH_3_ aliphatic chain); 1.25 (br s, 20 H, CH_2_ aliphatic chain); 1.42 (m, 2 H, CH_2_ aliphatic chain); 1.78 (q, 2 H, ^3^*J* (H,H) = 6.9 Hz, O–CH_2_–CH_2_); 3.92 (t, 2 H, ^3^*J* (H,H) = 6.5 Hz, OCH_2_); 4.07 (s, 3 H, CH_3_–N); 5.48 (s, 2 H, N–CH_2_–Ph); 6.88 (d, 2 H, ^3^*J* (H,H) = 8.6 Hz, CH phenyl); 7.23 (s, 1 H, CH imidazolium); 7.37 (s, 1 H, CH imidazolium); 7.41 (d, 2 H, ^3^*J* (H,H) = 8.6 Hz, CH phenyl); 10.50 (s, 1 H, N–CH–N).

^13^C NMR (75 MHz; CDCl_3_ {H})

δ: 14.05 (CH_3_ aliphatic chain); 22.63; 25.98; 29.14; 29.30; 29.35; 29.53; 29.56; 29.62; 31.87 (CH_2_ aliphatic chain); 36.75 (N–CH_3_); 53.08 (N–CH_2_–Ph); 68.19 (O–CH_2_); 115.33 (CH phenyl); 121.45; 123.14 (CH imidazolium); 124.41 (C phenyl); 130.62 (CH phenyl); 137.55 (N–CH–N); 160.12 (C phenyl).

IR: ν_max_/cm^−1^  3440 (H_2_O) 3133 (C–H aromatic), 2919 and 2850 (C–H aliphatic), 1513 (C=C aromatic), 1249 (aromatic ether).

Elemental analysis (%) calcd for C_25_H_41_BrN_2_O·H_2_O: C, 62.1; H, 9.0; N, 5.8; found: C, 61.9; H, 8.9; N, 5.7

**2_14_: 1-(4-Tetradecyloxybenzyl)-3-methyl-1*H*-imidazol-3-ium tetrafluoroborate**

Sodium tetrafluoroborate: 0.12 g (1.07 mmol)

1-(4-Tetradecyloxybenzyl)-3-methyl-1*H*-imidazol-3-ium bromide: 0.5 g (1.07 mmol)

Water: 60 mL

Yield: 0.434 g, 0.92 mmol (86%)

^1^H NMR (300 MHz; CDCl_3_)

δ: 0.88 (t, 3 H, ^3^*J* (H,H) = 6.9 Hz, CH_3_ aliphatic chain); 1.26 (br s, 20 H, CH_2_ aliphatic chain); 1.42 (m, 2 H, CH_2_ aliphatic chain); 1.76 (q, 2 H, ^3^*J* (H,H) = 6.9 Hz, O–CH_2_–CH_2_); 3.89–3.93 (m, 5H, CH_3_–N, OCH_2_); 5.24 (s, 2 H, N–CH_2_–Ph); 6.89 (d, 2 H, ^3^*J* (H,H) = 8.6 Hz, CH phenyl); 7.16 (s, 1 H, CH imidazolium); 7.26 (s, 1 H, CH imidazolium); 7.32 (d, 2 H, ^3^*J* (H,H) = 8.6 Hz, CH phenyl); 8.87 (s, 1 H, N–CH–N).

^13^C NMR (75 MHz; CDCl_3_ {H})

δ: 14.07 (CH_3_ aliphatic chain); 22.66; 26.01; 29.18; 29.33; 29.39; 29.57; 29.59; 29.63; 29.66; 31.90 (CH_2_ aliphatic chain); 36.34 (N–CH_3_); 53.12 (N–CH_2_–Ph); 68.19 (O–CH_2_); 115.36 (CH phenyl); 121.62; 123.47 (CH imidazolium); 124.28 (C phenyl); 130.59 (CH phenyl); 136.47 (N–CH–N); 160.14 (C phenyl).

IR: ν_max_/cm^−1^  3157 (C–H aromatic), 2917 and 2850 (C–H aliphatic), 1515 (C=C aromatic), 1251 (aromatic ether), 1051 (BF_4_^−^)

Elemental analysis (%) calcd for C_25_H_41_BF_4_N_2_O: C, 63.6; H, 8.8; N, 5.9; found: C, 63.6; H, 8.8; N, 5.85

**3_14_: 1-(4-Tetradecyloxybenzyl)-3-methyl-1*H*-imidazol-3-ium hexafluorophosphate**

Potassium hexafluorophosphate: 0.202 g (1.1 mmol)

1-(4-Tetradecyloxybenzyl)-3-methyl-1*H*-imidazol-3-ium bromide: 0.5 g (1.1 mmol)

Water: 60 mL

Yield: 0.51 g, 0.96 mmol (87%)

^1^H NMR (300 MHz; CDCl_3_)

δ: 0.88 (t, 3 H, ^3^*J* (H,H) = 6.9 Hz, CH_3_ aliphatic chain); 1.25 (br s, 20 H, CH_2_ aliphatic chain); 1.42 (m, 2 H, CH_2_ aliphatic chain); 1.76 (q, 2 H, ^3^*J* (H,H) = 6.9 Hz, O–CH_2_–CH_2_); 3.86 (s, 3 H, CH_3_–N); 3.92 (t, 2 H, ^3^*J* (H,H) = 6.5 Hz , OCH_2_); 5.18 (s, 2 H, N–CH_2_–Ph); 6.89 (d, 2 H, ^3^*J* (H,H) = 8.6 Hz, CH phenyl); 7.12 (s, 1 H, CH imidazolium); 7.21 (s, 1 H, CH imidazolium); 7.29 (d, 2 H, ^3^*J* (H,H) = 8.6 Hz, CH phenyl); 8.52 (s, 1 H, N–CH–N).

^13^C NMR (75 MHz; CDCl_3_ {H})

δ: 14.10 (CH_3_ aliphatic chain); 22.67; 26.01; 29.17; 29.34; 29.41; 29.67; 31.90 (CH_2_ aliphatic chain); 36.23 (N–CH_3_); 53.11 (N–CH_2_–Ph); 68.17 (O–CH_2_); 115.37 (CH phenyl); 121.64; 123.51 (CH imidazolium); 123.91 (C phenyl); 130.59 (CH phenyl); 135.85 (N–CH–N); 160.15 (C phenyl).

IR: ν_max_/cm^−1^  3179 (C–H aromatic), 2916 and 2849 (C–H aliphatic), 1517 (C=C aromatic), 1252 (aromatic ether), 824 (PF_6_^−^).

Elemental analysis (%) calcd for C_25_H_41_F_6_N_2_OP: C, 56.6; H, 7.8; N, 5.3; found: C, 56.9; H, 7.7; N, 5.2

**4_14_: 1-(4-Tetradecyloxybenzyl)-3-methyl-1*H*-imidazol-3-ium thiocyanate**

Potassium thiocyanate: 0.208 g (2.14 mmol)

1-(4-Tetradecyloxybenzyl)-3-methyl-1*H*-imidazol-3-ium bromide: 0.500 g (1.07 mmol)

Water: 60 mL

Yield: 403 mg, 0.91 mmol (85%)

^1^H NMR (300 MHz; CDCl_3_)

δ: 0.88 (t, 3 H, ^3^*J* (H,H) = 6.9 Hz, CH_3_ aliphatic chain); 1.26 (br s, 20 H, CH_2_ aliphatic chain); 1.42 (m, 2 H, CH_2_ aliphatic chain); 1.77 (q, 2 H, ^3^*J* (H,H) = 6.9 Hz, O–CH_2_–CH_2_); 3.93 (t, 2 H, ^3^*J* (H,H) = 6.5 Hz , OCH_2_); 4.06 (s, 3 H, CH_3_–N); 5.42 (s, 2 H, N–CH_2_–Ph); 6.91 (d, 2 H, ^3^*J* (H,H) = 8.6 Hz, CH phenyl); 7.26 (s, 1 H, CH imidazolium); 7.33 (s, 1 H, CH imidazolium); 7.39 (d, 2 H, ^3^*J* (H,H) = 8.6 Hz, CH phenyl); 9.48 (s, 1 H, N–CH–N).

^13^C NMR (75 MHz; CDCl_3_ {H})

δ: 14.06 (CH_3_ aliphatic chain); 22.64; 25.99; 29.15; 29.31; 29.36; 29.54; 29.57; 29. 61; 29.63; 31.88 (CH_2_ aliphatic chain); 36.82 (N–CH_3_); 53.45 (N–CH_2_–Ph); 68.21 (O–CH_2_); 115.41 (CH phenyl); 121.76; 123.43 (CH imidazolium); 124.10 (C phenyl); 130.71 (CH phenyl); 131.73 (SCN); 137.00 (N–CH–N); 160.20 (C phenyl).

IR: ν_max_/cm^−1^  3350 (H_2_O) 3147 (C–H aromatic), 2917 and 2850 (C–H aliphatic), 2065 (SCN^−^) 1515 (C=C aromatic), 1251 (aromatic ether).

Elemental analysis (%) calcd for C_26_H_41_N_3_OS·¾ H_2_O: C, 68.3; H, 9.4; N, 9.2; found: C, 68.2; H, 9.2; N, 9.1

**5_14_: 1-(4-Tetradecyloxybenzyl)-3-methyl-1*H*-imidazol-3-ium trifluoromethanesulfonate**

Sodium trifluoromethanesulfonate: 0.368 g (2.14 mmol)

1-(4-Tetradecyloxybenzyl)-3-methyl-1*H*-imidazol-3-ium bromide: 0.5 g (1.07 mmol)

water: 60 mL

Yield: 0.4 g, 0.75 mmol (70%)

^1^H NMR (300 MHz; CDCl_3_)

δ: 0.89 (t, 3 H, ^3^*J* (H,H) = 6.8 Hz, CH_3_ aliphatic chain); 1.27 (br s, 16 H, CH_2_ aliphatic chain); 1.41 (m, 2 H, CH_2_ aliphatic chain); 1.78 (q, 2 H, ^3^*J* (H,H) = 6.6 Hz, O–CH_2_–CH_2_); 3.95 (t, 2 H, ^3^*J* (H,H) = 6.6 Hz, OCH_2_); 3.99 (s, 3 H, CH_3_–N); 5.31 (s, 2 H, N–CH_2_–Ph); 6.92 (d, 2 H, ^3^*J* (H,H) = 8.6 Hz, CH phenyl); 7.10 (m, 1 H, CH imidazolium); 7.16 (m, 1 H, CH imidazolium); 7.32 (d, 2 H, ^3^*J* (H,H) = 8.6 Hz, CH phenyl); 9.40 (s, 1 H, N–CH–N).

^13^C NMR (75 MHz; CDCl_3_ {H})

δ: 14.02 (CH_3_ aliphatic chain); 22.60; 25.94; 29.10; 29.26; 29.26; 29.32; 29.50; 29.53; 29.58; 31.83 (CH_2_ aliphatic chain); 36.26 (N–CH_3_); 52.96 (N–CH_2_–Ph); 68.10(O–CH_2_); 115.23 (CH phenyl); 120.6 (q, *J (*C–F)= 320 Hz, CF_3_); 121.76; 123.61 (CH imidazolium); 124.31 (C phenyl); 130.46 (CH phenyl); 136.34 (N–CH–N); 160.02 (C phenyl).

IR: ν_max_/cm^−1^  3157 (C–H aromatic), 2918 and 2849 (C–H aliphatic), 1517 (C=C aromatic), 1251 and 1030 (CF_3_SO_3_^−^).

Elemental analysis (%) calcd for C_26_H_41_F_3_N_2_O_4_S: C, 58.4; H, 7.7; N, 5.2; found: C, 58.25; H, 7.6; N, 5.2

**6_14_: 1-(4-Tetradecyloxybenzyl)-3-methyl-1*H*-imidazol-3-ium bis[(trifluoromethyl)sulfonyl]amide**

Lithium bis[(trifluoromethyl)sulfonyl]amide: 0.171 g (0.59 mmol)

1-(4-Tetradecyloxybenzyl)-3-methyl-1*H*-imidazol-3-ium bromide: 0.25 g (0.54 mmol)

Water: 30 mL

Yield: 0.275 g, 0.41 mmol (76%)

^1^H NMR (300 MHz; CDCl_3_)

δ: 0.89 (t, 3 H, ^3^*J* (H,H) = 6.4 Hz, CH_3_ aliphatic chain); 1.27 (br s, 20 H, CH_2_ aliphatic chain); 1.44 (m, 2 H, CH_2_ aliphatic chain); 1.78 (q, 2 H, ^3^*J* (H,H) = 6.6 Hz, O–CH_2_–CH_2_); 3.93 (s, 3 H, CH_3_–N); 3.96 (t, 2 H, ^3^*J* (H,H) = 6.6 Hz , OCH_2_); 5.25 (s, 2 H, N–CH_2_–Ph); 6.92 (d, 2 H, ^3^*J* (H,H) = 8.8 Hz, CH phenyl); 7.15 (s, 1 H, CH imidazolium); 7.22 (s, 1 H, CH imidazolium); 7.29 (d, 2 H, ^3^*J* (H,H) = 8.6 Hz, CH phenyl); 8.79 (s, 1 H, N–CH–N).

^13^C NMR (75 MHz; CDCl_3_ {H})

δ: 14.07 (CH_3_ aliphatic chain); 22.67; 26.00; 29.16; 29.33; 29.37; 29.56; 29.58; 29.66; 31.91 (CH_2_ aliphatic chain); 36.50 (N–CH_3_); 53.41 (N–CH_2_–Ph); 68.27 (O–CH_2_); 115.53 (CH phenyl); 119.83 (quadruplet, *J* (C,F)= 319.10 Hz, CF3); 121.80; 123.49 (CH imidazolium); 123.57 (C phenyl); 130.58 (CH phenyl); 136.12 (N–CH–N); 160.39 (C phenyl).

IR: ν_max_/cm^−1^  3157 (C–H aromatic), 2917 and 2848 (C–H aliphatic), 1517 (C=C aromatic), 1353 and 1181 ([CF_3_SO_2_]_2_N^−^)

Elemental analysis (%) calcd for C_27_H_41_F_6_N_3_O_5_S_2_·½ H_2_O: C, 48.1; H, 6.3; N, 6.2; found: C, 48.15; H, 6.1; N, 6.2

**1_16_: 1-(4-Hexadecyloxybenzyl)-3-methyl-1*H*-imidazol-3-ium bromide**

**A_16_**: 5 g (14.4 mmol)

Thionyl bromide: 1.3 mL (16.7 mmol)

Dichloromethane: 50 mL

*N*-Methylimidazole: 1.3 mL (15.8 mmol)

Tetrahydrofuran: 50 mL

Yield: 6.06 g, 12.2 mmol (85%)

^1^H NMR (300 MHz; CDCl_3_)

δ: 0.88 (t, 3 H, ^3^*J* (H,H) = 6.9 Hz, CH_3_ aliphatic chain); 1.26 (br s, 24 H, CH_2_ aliphatic chain); 1.43 (m, 2 H, CH_2_ aliphatic chain); 1.77 (q, 2 H, ^3^*J* (H,H) = 6.9 Hz, O–CH_2_–CH_2_); 3.91 (t, 2 H, ^3^*J* (H,H) = 6.5 Hz, OCH_2_); 4.08 (s, 3 H, CH_3_–N); 5.48 (s, 2 H, N–CH_2_–Ph); 6.89 (d, 2 H, ^3^*J* (H,H) = 8.6 Hz, CH phenyl); 7.18 (s, 1 H, CH imidazolium); 7.25 (s, 1 H, CH imidazolium); 7.40 (d, 2 H, ^3^*J* (H,H) = 8.6 Hz, CH phenyl); 10.56 (s, 1 H, N–CH–N).

^13^C NMR (75 MHz; CDCl_3_ {H})

δ: 14.04 (CH_3_ aliphatic chain); 22.61; 25.95; 29.10; 29.28; 29.33; 29.51; 31.84 (CH_2_ aliphatic chain); 36.74 (N–CH_3_); 52.96 (N–CH_2_–Ph); 68.11 (O–CH_2_); 115.21 (CH phenyl); 121.51; 123.26 (CH imidazolium); 124.47 (C phenyl); 130.59 (CH phenyl); 137.21 (N–CH–N); 159.99 (C phenyl).

IR: ν_max_/cm^−1^  3421 (H_2_O) 3133 (C–H aromatic), 2915 and 2849 (C–H aliphatic), 1515 (C=C aromatic), 1250 (aromatic ether).

Elemental analysis (%) calcd for C_27_H_45_BrN_2_O·H_2_O: C, 63.4; H, 9.3; N, 5.5; found: C, 63.3; H, 9.0; N, 5.4

**2_16_: 1-(4-Hexadecyloxybenzyl)-3-methyl-1*H*-imidazol-3-ium tetrafluoroborate**

Sodium tetrafluoroborate: 0.118 g (1.05 mmol)

1-(4-Hexadecyloxybenzyl)-3-methyl-1*H*-imidazol-3-ium bromide: 0.5 g (1.05 mmol)

Water: 60 mL

Yield: 0.5 g, 1.00 mmol (95%)

^1^H NMR (300 MHz; CDCl_3_)

δ: 0.88 (t, 3 H, ^3^*J* (H,H) = 6.9 Hz, CH_3_ aliphatic chain); 1.26 (br s, 24 H, CH_2_ aliphatic chain); 1.39–1.47 (m, 2 H, CH_2_ aliphatic chain); 1.76 (m, 2 H, O–CH_2_–CH_2_); 3.89–3.93 (m, 5H, CH_3_–N, OCH_2_); 5.23 (s, 2 H, N–CH_2_–Ph); 6.87 (d, 2 H, ^3^*J* (H,H) = 8.6 Hz, CH phenyl); 7.17 (m, 1 H, CH imidazolium); 7.27 (m, 1 H, CH imidazolium); 7.31 (d, 2 H, ^3^*J* (H,H) = 8.6 Hz, CH phenyl); 8.83 (s, 1 H, N–CH–N).

^13^C NMR (75 MHz; CDCl_3_ {H})

δ: 14.08 (CH_3_ aliphatic chain); 22.66; 26.00; 29.17; 29.33; 29.40; 29.57; 29.60; 29.62; 29.67; 31.89 (CH_2_ aliphatic chain); 36.26 (N–CH_3_); 53.00 (N–CH_2_–Ph); 68.16 (O–CH_2_); 115.29 (CH phenyl); 121.63; 123.52 (CH imidazolium); 124.36 (C phenyl); 130.57 (CH phenyl); 136.29 (N–CH–N); 160.06 (C phenyl).

IR: ν_max_/cm^−1^  3160 (C–H aromatic), 2916 and 2849 (C–H aliphatic), 1516 (C=C aromatic), 1251 (aromatic ether), 1048 (BF_4_^−^)

Elemental analysis (%) calcd for C_27_H_45_BF_4_N_2_O·¾ H_2_O: C, 63.1; H, 9.1; N, 5.45; found: C, 63.0; H, 9.0; N, 5.4

**3_16_: 1-(4-Hexadecyloxybenzyl)-3-methyl-1*H*-imidazol-3-ium hexafluorophosphate**

Potassium hexafluorophosphate: 0.181 g (0.98 mmol)

1-(4-Hexadecyloxybenzyl)-3-methyl-1*H*-imidazol-3-ium bromide: 0.5 g (0.98 mmol)

Water: 60 mL

Yield: 0.472 g, 0.84 mmol (86%)

^1^H NMR (300 MHz; CDCl_3_)

δ: 0.88 (t, 3 H, ^3^*J* (H,H) = 6.9 Hz, CH_3_ aliphatic chain); 1.26 (br s, 24 H, CH_2_ aliphatic chain); 1.44 (m, 2 H, CH_2_ aliphatic chain); 1.76 (q, 2 H, ^3^*J* (H,H) = 6.6 Hz, O–CH_2_–CH_2_); 3.88 (s, 3 H, CH_3_–N); 3.90 (t, 2 H, ^3^*J* (H,H) = 6.5 Hz , OCH_2_); 5.20 (s, 2 H, N–CH_2_–Ph); 6.90 (d, 2 H, ^3^*J* (H,H) = 8.6 Hz, CH phenyl); 7.12 (s, 1 H, CH imidazolium); 7.21 (s, 1 H, CH imidazolium); 7.30 (d, 2 H, ^3^*J* (H,H) = 8.6 Hz, CH phenyl); 8.59 (s, 1 H, N–CH–N).

^13^C NMR (75 MHz; CDCl_3_ {H})

δ: 14.10 (CH_3_ aliphatic chain); 22.67; 26.01; 29.17; 29.34; 29.41; 29.58; 29.62; 29.64; 29.68; 31.91 (CH_2_ aliphatic chain); 36.27 (N–CH_3_); 53.14 (N–CH_2_–Ph); 68.18 (O–CH_2_); 115.37 (CH phenyl); 121.62; 123.47 (CH imidazolium); 123.89 (C phenyl); 130.59 (CH phenyl); 135.95 (N–CH–N); 160.16 (C phenyl).

IR: ν_max_/cm^−1^  3154 (C–H aromatic), 2916 and 2850 (C–H aliphatic), 1517 (C=C aromatic), 1252 (aromatic ether), 825 (PF_6_^−^)

Elemental analysis (%) calcd for C_27_H_45_F_6_N_2_OP: C, 58.05; H, 8.1; N, 5.0; found: C, 58.2; H, 8.0; N, 5.0

**4_16_: 1-(4-Hexadecyloxybenzyl)-3-methyl-1*H*-imidazol-3-ium thiocyanate**

Potassium thiocyanate: 0.196 g (2.02 mmol)

1-(4-Tetradecyloxybenzyl)-3-methyl-1*H*-imidazol-3-ium bromide: 0.5 g (1.01 mmol)

Water: 60 mL

Yield: 0.395 g, 0.83 mmol (83%)

^1^H NMR (300 MHz; CDCl_3_)

δ: 0.88 (t, 3 H, ^3^*J* (H,H) = 6.9 Hz, CH_3_ aliphatic chain); 1.26 (br s, 24 H, CH_2_ aliphatic chain); 1.44 (m, 2 H, CH_2_ aliphatic chain); 1.77 (q, 2 H, ^3^*J* (H,H) = 6.6 Hz, O–CH_2_–CH_2_); 3.93 (t, 2 H, ^3^*J* (H,H) = 6.5 Hz , OCH_2_); 4.06 (s, 3 H, CH_3_–N); 5.42 (s, 2 H, N–CH_2_–Ph); 6.90 (d, 2 H, ^3^*J* (H,H) = 8.6 Hz, CH phenyl); 7.25 (m, 1 H, CH imidazolium); 7.34 (m, 1 H, CH imidazolium); 7.38 (d, 2 H, ^3^*J* (H,H) = 8.6 Hz, CH phenyl); 9.47 (s, 1 H, N–CH–N).

^13^C NMR (75 MHz; CDCl_3_ {H})

δ: 14.06 (CH_3_ aliphatic chain); 22.64; 25.99; 29.15; 29.31; 29.37; 29.54; 29.57; 29.65; 31.89 (CH_2_ aliphatic chain); 36.81 (N–CH_3_); 53.43 (N–CH_2_–Ph); 68.21 (O–CH_2_); 115.42 (CH phenyl); 121.77; 123.45 (CH imidazolium); 124.13 (C phenyl); 130.70 (CH phenyl); 131.70 (SCN); 136.97 (N–CH–N); 160.18 (C phenyl).

IR: ν_max_/cm^−1^  3147 (C–H aromatic); 2916 and 2849 (C–H aliphatic); 2064 (SCN), 1515 (C=C aromatic); 1249 (aromatic ether)

Elemental analysis (%) calcd for C_28_H_45_N_3_OS + H_2_O: C, 68.7; H, 9.7; N, 8.6; found: C, 68.7; H, 9.6; N, 8.6

**5_16_: 1-(4-Hexadecyloxybenzyl)-3-methyl-1*H*-imidazol-3-ium trifluoromethanesulfonate**

Sodium trifluoromethanesulfonate: 0.348 g (2.02 mmol)

1-(4-Hexadecyloxybenzyl)-3-methyl-1*H*-imidazol-3-ium bromide: 0.5 g (1.01 mmol)

Water: 60 mL

Yield: 0.41 g, 0.72 mmol (72%)

^1^H NMR (300 MHz; CDCl_3_)

δ: 0.88 (t, 3 H, ^3^*J* (H,H) = 6.8 Hz, CH_3_ aliphatic chain); 1.26 (br s, 24 H, CH_2_ aliphatic chain); 1.41 (m, 2 H, CH_2_ aliphatic chain); 1.77 (q, 2 H, ^3^*J* (H,H) = 6.6 Hz, O–CH_2_–CH_2_); 3.92 (t, 2 H, ^3^*J* (H,H) = 6.6 Hz, OCH_2_); 3.95 (s, 3 H, CH_3_–N); 5.26 (s, 2 H, N–CH_2_–Ph); 6.89 (d, 2 H, ^3^*J* (H,H) = 8.6 Hz, CH phenyl); 7.21 (m, 1 H, CH imidazolium); 7.29 (m, 1 H, CH imidazolium); 7.32 (d, 2 H, ^3^*J* (H,H) = 8.6 Hz, CH phenyl); 9.14 (s, 1 H, N–CH–N).

^13^C NMR (75 MHz; CDCl_3_ {H})

δ: 14.07 (CH_3_ aliphatic chain); 22.65; 26.00; 29.17; 29.31; 29.38; 29.56; 29.59; 29.66; 31.89 (CH_2_ aliphatic chain); 36.38 (N–CH_3_); 53.14 (N–CH_2_–Ph); 68.19 (O–CH_2_); 115.37 (CH phenyl); 120.7 (q, *J (*C–F)= 320 Hz, CF_3_); 121.71; 123.50 (CH imidazolium); 124.21 (C phenyl); 130.54 (CH phenyl); 136.68 (N–CH–N); 160.17 (C phenyl).

IR: ν_max_/cm^−1^  3122 (C–H aromatic), 2918 and 2849 (C–H aliphatic), 1516 (C=C aromatic), 1260 and 1030 (CF_3_SO_3_^−^).

Elemental analysis (%) calcd for C_28_H_45_F_3_N_2_O_4_S: C, 59.8; H, 8.1; N, 5.0; found: C, 60.3; H, 8.2; N, 4.9

**6_16_: 1-(4-Hexadecyloxybenzyl)-3-methyl-1*H*-imidazol-3-ium bis[(trifluoromethyl)sulfonyl]amide**

Lithium bis[(trifluoromethyl)sulfonyl]amide: 0.161 g (0.56 mmol)

1-(4-Tetradecyloxybenzyl)-3-methyl-1*H*-imidazol-3-ium bromide: 0.25 g (0.51 mmol)

Water: 30 mL

Yield: 0.285 g, 0.41 mmol (81%)

^1^H NMR (300 MHz; CDCl_3_)

δ: 0.89 (t, 3 H, ^3^*J* (H,H) = 6.7 Hz, CH_3_ aliphatic chain); 1.27 (br s, 24 H, CH_2_ aliphatic chain); 1.44 (m, 2 H, CH_2_ aliphatic chain); 1.78 (q, 2 H, ^3^*J* (H,H) = 6.6 Hz, O–CH_2_–CH_2_); 3.93 (s, 3 H, CH_3_–N); 3.95 (t, 2 H, ^3^*J* (H,H) = 6.5 Hz , OCH_2_); 5.25 (s, 2 H, N–CH_2_–Ph); 6.92 (d, 2 H, ^3^*J* (H,H) = 8.6 Hz, CH phenyl); 7.15 (s, 1 H, CH imidazolium); 7.22 (s, 1 H, CH imidazolium); 7.30 (d, 2 H, ^3^*J* (H,H) = 8.6 Hz, CH phenyl); 8.77 (s, 1 H, N–CH–N).

^13^C NMR (75 MHz; CDCl_3_ {H})

δ: 14.07 (CH_3_ aliphatic chain); 22.66; 26.01; 29.17; 29.34; 29.38; 29.57; 29.59; 29.67; 31.91 (CH_2_ aliphatic chain); 36.47 (N–CH_3_); 53.38 (N–CH_2_–Ph); 68.25 (O–CH_2_); 115.50 (CH phenyl); 119.83 (quadruplet, *J* (C,F)= 319.10 Hz, CF3); 121.81; 123.52 (CH imidazolium); 123.59 (C phenyl); 130.57 (CH phenyl); 136.06 (N–CH–N); 160.36 (C phenyl).

IR: ν_max_/cm^−1^  3156 (C–H aromatic), 2917 and 2848 (C–H aliphatic), 1517 (C=C aromatic), 1353 and 1180 ([CF_3_SO_2_]_2_N^−^)

Elemental analysis (%) calcd for C_29_H_45_F_6_N_3_O_5_S_2_: C, 50.2; H, 6.5; N, 6.1; found: C, 50.4; H, 6.7; N, 5.9

Liquid Crystalline properties.

**Table SI-1:** Temperatures and enthalpy changes of the phase transitions for **1_12–16_** to **6_12–16_**.

Cr: solid; SmA: Smectic A phase; I: isotropic liquid; Dec: decomposition.

| **1_n_**–**5_n_** | Phase sequences  °C (kJ·mol^−1^ ) | **2_n_**–**6_n_** | Phase sequences  °C (kJ·mol^−1^ ) |
| --- | --- | --- | --- |
| **1_12_** | Cr_1_ −18.2(13.4) Cr_2_ 30.4(35.6) SmA 250(–)^b^ Iso  Iso 250(–)^b^ SmA 9.7(12.6) Cr_1_ | **2_12_** | Cr_1_ 13.8(11.9)^a^ Cr_2_ 61.5(29.6) Cr_3_ 65(2.5) SmA 203 (–)^b^ Iso  Iso 200(–)^b^ SmA 11(18.2) Cr_1_ |
| **3_12_** | Cr_1_ 16.7 (7.6)^a^ Cr_2_ 75.4(41) SmA 148(0.6) Iso  Iso 147.5(0.6) SmA 11.3(14.5) Cr_1_ | **4_12_** | Cr 52.7(48.3) SmA 188(0.9) I  I 188(0.9) SmA 34.4(50.0) Cr |
| **5_12_** | Cr 67(48.1) I  I 63.5(0.4) SmA 28(40.5) Cr | **6_12_** | Cr 49.5(60.5) I  I 20.3(49.5) Cr |
| **1_14_** | Cr 32.9(18.5) SmA 250(–) Dec  SmA 24.4(20.0) Cr | **2_14_** | Cr_1_ 35.2(18.9) Cr_2_ 49(16.8)^a^ Cr_3_  56(7.4) Cr_4_71.6(13.4) SmA 250 (–)Dec  SmA 32.5(23.1) Cr |
| **3_14_** | Cr_1_ 35.6(8.0)^b^ Cr_2_ 81(42.6) SmA 195(0.9) Iso  Iso194.2(0.9) SmA 31.7(21.7) Cr | **4_14_** | Cr 52(29.6) SmA 250(–) Dec  SmA 34.3(36.5) Cr |
| **5_14_** | Cr_1_ 24.4(19.7) ^b^ Cr_2_ 74.1(47.9) SmA 111.3(0.7) Iso  Iso 110.7(1.4) SmA 33(24.0) | **6_14_** | Cr 61,7(69.2) Iso  Iso 33,4(62.9) Cr |
| **1_16_** | Cr 48.2(20.7) SmA 250(–) Dec  SmA 44.8(28.8) Cr | **2_16_** | Cr_1_ 50.6(23.0) Cr_2_ 59(39.1) ^a^ Cr_3_ 77.7(40.7) SmA  250 (–) Dec  SmA 49(25.25) Cr_1_ |
| **3_16_** | Cr_1_ 45.4(24.15)^a^ Cr_2_ 86.9(55.4)  SmA 250(–) Dec  SmA 48.1(27.7) Cr | **4_16_** | Cr 62(45.6) SmA 250(–) Dec  SmA 45,3(38.5) Cr |
| **5_16_** | Cr_1_ 40,9(25.8)^a^ Cr_2_ 79,6(58.7)  SmA 148(1.45) Iso  Iso 147.6(29.5) SmA 48.1(1.2) Cr | **6_16_** | Cr 56.3(73.3) Iso  Iso 23.9(61.0) Cr |

^a^Cold crystallisation, ^b^Transition observed by POM

**Table SI-2:** Indexation of the reflections detected in the SmA liquid-crystalline phase by SAXS/WAXS for **1_8_**–**4_8_** at given temperatures. *d*_hkl_ (mes) are the measured diffraction spacing and correspond to the periodicity of layer. *I* corresponds to the intensity of the reflections (++: strong, +: weak).

| Compounds | *T* (°C) | *d*_00l(mes)_(Å) | *I* (u.a.) | 00l |
| --- | --- | --- | --- | --- |
| **1_8_** | 100 | 31.73 | ++ | 001 |
| **2_8_** | 30 | 32.88 | ++ | 001 |
| **4_8_** | 30 | 32.77 | ++ | 001 |

**Table SI-3:** Indexation of the reflections detected in the SmA liquid-crystalline phase by SAXS/WAXS for **1_10_**–**4_10_** at given temperatures. *d*_00l(mes)_ and *d*_00l(calc)_ are the measured and calculated diffraction spacing, d_001_ is the periodicity of the smectic phase *d*_00l(calc)_ = (2·*d*_002(mes)_ + *d*_001(mes)_)/2. I corresponds to the intensity of the reflections (++: strong, +: weak).

| Compounds | *T* (°C) | *d*_00l(mes)_(Å) | I (u.a.) | 00l | *d*_00l(calc)_(Å) |
| --- | --- | --- | --- | --- | --- |
| **1_10_** | 80 | 34.89  17.38 | ++  + | 001  002 | 34.82  17.41 |
| **2_10_** | 80 | 33,91  17.02 | ++  + | 001  002 | 33.98  16.99 |
| **3_10_** | 80 | 33.48 | ++ | 001 | 33.48 |
| **4_10_** | 80 | 34.08  17.02 | ++  + | 001  002 | 34.06  17.03 |

**Table SI-4:** Indexation of the reflections detected in the SmA liquid-crystalline phase by SAXS/WAXS for **1_12_**–**4_12_** at given temperatures. *d*_00l(mes)_ and *d*_00l(calc)_ are the measured and calculated diffraction spacing, *d*_001_ is the periodicity of the smectic phase *d*_00l(calc)_ = (2·*d*_002(mes)_ + *d*_001(mes)_)/2. I corresponds to the intensity of the reflections (++: strong, +: weak).

| Compounds | *T* (°C) | *d*_00l(mes)_(Å) | I (u.a.) | 00l | *d*_00l(calc)_(Å) |
| --- | --- | --- | --- | --- | --- |
| **1_12_** | 100 | 37.45 | ++ | 001 | 37.45 |
| **2_12_** | 100 | 36.07  17.94  12.02 | ++  +  + | 001  002  003 | 35.95  17.98  11.98 |
| **3_12_** | 100 | 35.35 | ++ | 001 | 35.35 |
| **4_12_** | 100 | 36.03  17.85 | ++  + | 001  002 | 35.86  17.93 |

**Table SI-5:** Indexation of the reflections detected in the SmA liquid-crystalline phase by SAXS/WAXS for **1_14_**–**5_14_** at given temperatures. d_00l(mes)_ and d_00l(calc)_ are the measured and calculated diffraction spacing, d_001_ is the periodicity of the smectic phase d_00l(calc)_ = (2·*d*_002(mes)_ + *d*_001(mes)_)/2. I corresponds to the intensity of the reflections (++: strong, +: weak).

| Compounds | *T* (°C) | *d*_00l(mes)_(Å) | *I* (u.a.) | 00l | *d*_00l(calc)_(Å) |
| --- | --- | --- | --- | --- | --- |
| **1_14_** | 100 | 40.29  20.01 | ++  + | 001  002 | 40.15  20.08 |
| **2_14_** | 100 | 39.09 | ++ | 001 | 39.09 |
| **3_14_** | 100 | 38.08  18.97 | ++  + | 001  002 | 38.01  19.00 |
| **4_14_** | 100 | 38.54  19.30 | ++  + | 001  003 | 38.68  19.34 |
| **5_14_** | 100 | 35.93 | ++ | 001 | 35.93 |

**Table SI-6:** Indexation of the reflections detected in the SmA liquid-crystalline phase by SAXS/WAXS for **1_16_**–**5_16_** at given temperatures. *d*_00l(mes)_ and *d*_00l(calc)_ are the measured and calculated diffraction spacing, d_001_ is the periodicity of the smectic phase *d*_00l(calc)_ = (2·*d*_002(mes)_ + *d*_001(mes)_)/2. *I* corresponds to the intensity of the reflections (++: strong, +: weak).

| Compounds | *T* (°C) | *d*_00l (mes)_ **/** Å | *I* (u.a.) | 00l | *d*_00l(cal)_ **/** Å |
| --- | --- | --- | --- | --- | --- |
| **1_16_** | 100 | 43.72  21.68 | ++  + | 001  002 | 43.54  21.77 |
| **2_16_** | 100 | 41.67  20.87 | ++  + | 001  002 | 41.71  20.85 |
| **3_16_** | 100 | 40.53  20.24 | ++  + | 001  002 | 40.51  20.26 |
| **4_16_** | 100 | 41.41  13.67 | ++  + | 001  003 | 41.21  13.74 |
| **5_16_** | 100 | 38.53  18.91 | ++  + | 001  002 | 38.18  19.09 |

**Table SI-7:** Temperature of the isotropic transition and molecular area limit calculated at the clearing point.

| Compounds | S_limit_ (Å^2^) | *T*_iso_ (°C) | Compounds | S_limit_ (Å^2^) | *T*_iso_ (°C) |
| --- | --- | --- | --- | --- | --- |
| **1_12_** | 43.6^a^ | 250 | **1_14_** | – | 263^b^ |
| **2_12_** | 44.1^a^ | 200 | **2_14_** | – | 248^b^ |
| **3_12_** | 43.2^a^ | 148 | **3_14_** | 46.2^a^ | 195 |
| **4_12_** | 42.9^a^ | 188 | **4_14_** | – | 233^b^ |
|  |  |  | **5_14_** | 47.9^a^ | 111 |

^a^Calculated according to the linear fit of the data obtained by the dilatometry study, ^b^calculated considering 47 Å^2^ as S_limit_


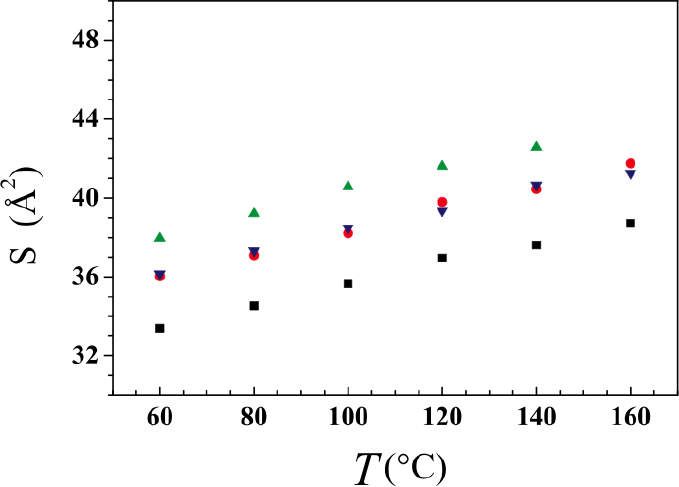


**Figure SI-1:** Variation with the counter-ion of the molecular area S in the smectic A phase for series: squares: **1_12_**; circles: **2_12_**; up triangles: **3_12_**; down triangles: **4_12_**.


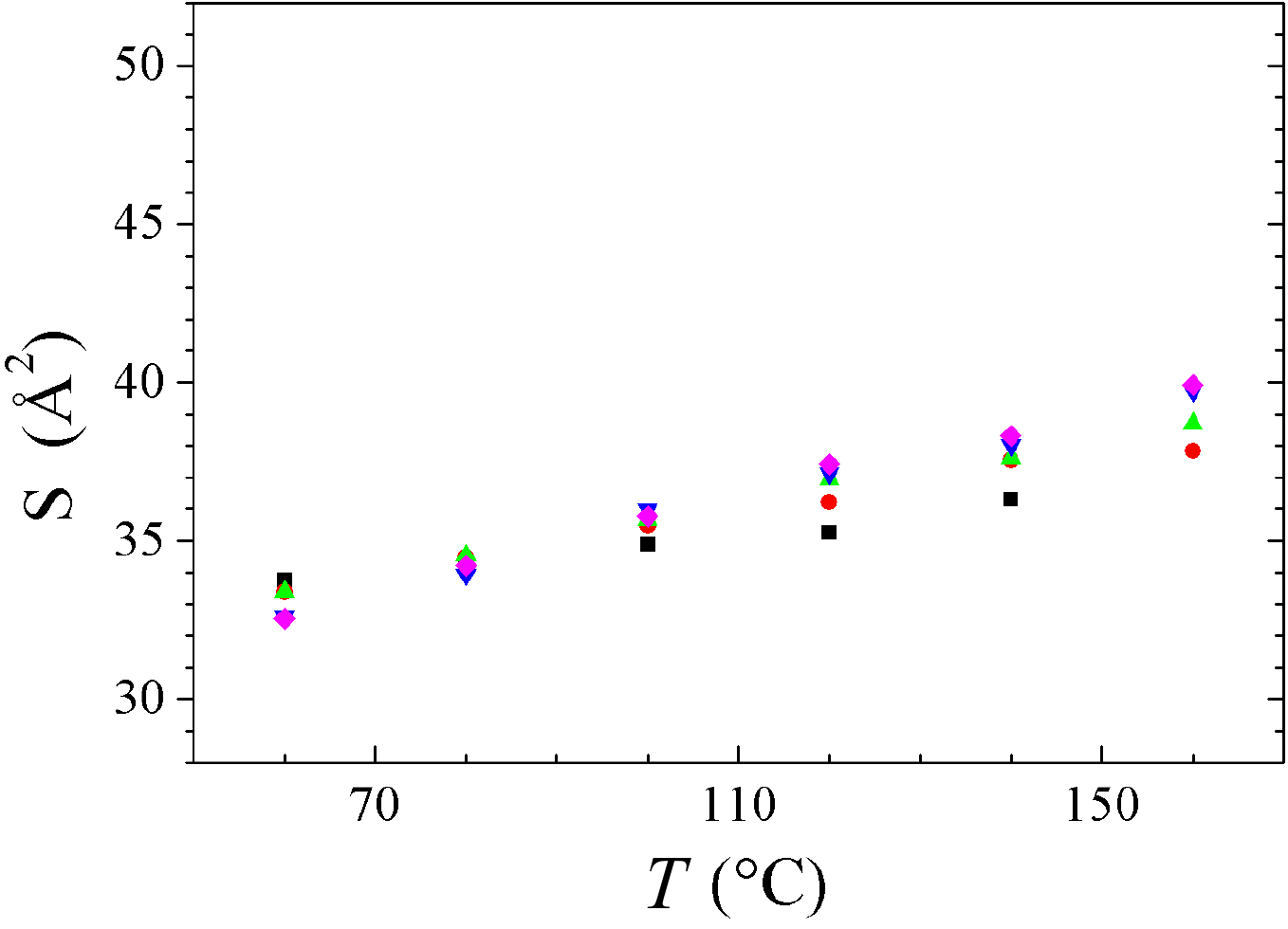


**Figure SI-2:** Variation with chain length of the molecular area S in the smectic A phase for the series with bromide anion: squares: **1_8_**; circles: **1_10_**; up triangles: **1_12_**; down triangles: **1_14_**; diamonds: **1_16_**.

1. the chemical shift is symbolised by δ and expressed in ppm [↑](#footnote-ref-2)
2. the multiplicity is symbolised by s: singlet; d: doublet; t: triplet; q: quintuplet and m: multiplet [↑](#footnote-ref-3)
3. the coupling constants are symbolised by *J* and are expressed in Hz [↑](#footnote-ref-4)
